# Sarcoidosis in an Italian province. Prevalence and environmental risk factors

**DOI:** 10.1371/journal.pone.0176859

**Published:** 2017-05-05

**Authors:** Deborah Beghè, Luca Dall’Asta, Claudia Garavelli, Augusto Alberto Pastorelli, Marilena Muscarella, Gloria Saccani, Marina Aiello, Ernesto Crisafulli, Massimo Corradi, Paolo Stacchini, Alfredo Chetta, Giuseppina Bertorelli

**Affiliations:** 1 Department of Food and Drug, University of Parma, Parma, Italy; 2 Department of Applied Science and Technology, Polytechnic University of Turin, Turin, Italy; 3 Collegio Carlo Alberto, Moncalieri, Turin, Italy; 4 Department of Medicine and Surgery, Occupational Medicine Unit, University of Parma, Parma, Italy; 5 National Reference Laboratory for Heavy Metals in Food, Department of Food Safety and Veterinary Public Health, Istituto Superiore di Sanità, Rome, Italy; 6 Istituto Zooprofilattico Sperimentale della Puglia e Basilicata, Foggia, Italy; 7 Department of Medicine and Surgery, University of Parma, Parma, Italy; 8 Department of Medicine and Surgery, Respiratory Disease and Lung Function Unit, University of Parma, Parma, Italy; Universitatsklinikum Freiburg, GERMANY

## Abstract

**Background and aim:**

Sarcoidosis is a systemic granulomatous inflammatory disease whose causes are still unknown and for which epidemiological data are often discordant. The aim of our study is to investigate prevalence and spatial distribution of cases, and identify environmental exposures associated with sarcoidosis in an Italian province.

**Methods:**

After georeferentiation of cases, the area under study was subdivided with respect to Municipality and Health Districts and to the altitude in order to identify zonal differences in prevalence. The bioaccumulation levels of 12 metals in lichen tissues were analyzed, in order to determine sources of air pollution. Finally, the analysis of the correlation between metals and between pickup stations was performed.

**Results:**

223 patients were identified (58.3% female and 41.7% male of total) and the mean age was 50.6±15.4 years (53.5±15.5 years for the females and 46.5±14.4 for the males). The mean prevalence was 49 per 100.000 individuals. However, we observed very heterogeneous prevalence in the area under study. The correlations among metals revealed different deposition patterns in lowland area respect to hilly and mountain areas.

**Conclusions:**

The study highlights a high prevalence of sarcoidosis cases, characterized by a very inhomogeneous and patchy distribution with phenomena of local aggregation. Moreover, the bioaccumulation analysis was an effective method to identify the mineral particles that mostly contribute to air pollution in the different areas, but it was not sufficient to establish a clear correlation between the onset of sarcoidosis and environmental risk factors.

## Introduction

Sarcoidosis is a systemic inflammatory disease of unknown etiology characterized by the accumulation of immune effector cells in affected organs along with the presence of non-caseating granulomas. It is considered a multifactorial disease likely to result from the interaction of environmental factors and multiple genes. The pathogenetic hypothesis of sarcoidosis involves an instigating antigen that is presented to T-lymphocytes. This results in the activation of CD4+ Th1 cells and a final pathway of aberrant granulomatous inflammation with aggregations of activated T cells and macrophages in areas of chronic inflammation [1–3].

The recent review of Valeyre et al. [3] estimated that the prevalence of this disease is between 4.7–64 cases per 100.000 individuals and the incidence is 1.0–35.5 in 100.000 per year. The highest rates are reported in northern European and African-American individuals, particularly in women, while are the lowest in Japan. Epidemiological data currently available are discordant. Different methods (*e*.*g*. surveys, databases, national registries, radiological screening programs, etc.) are used to calculate the incidence and prevalence of sarcoidosis in specific areas, depending on the age, gender, ethnic groups, and geographical location. Moreover, sarcoidosis is not always recognized and diagnosed; this difficulty is due to the variability in the clinical presentation, the absence of mass screening programs and the presence, in some geographical areas, of most commonly recognized granulomatous diseases (*e*.*g*. tuberculosis, leprosy, berylliosis, fungal infections) that may mask the diagnosis of sarcoidosis [4, 5]. Consequently, it is not straightforward to combine all existing epidemiologic information to obtain a clear knowledge of the overall prevalence or incidence of the disease.

In Italy, the first project with the aim to create an Italian Register on Diffuse Infiltrative Lung Disorders (RIPID-1), including sarcoidosis, was established in 1998, as a joint project of the major Italian Scientific Societies for Respiratory Medicine. The project has led to record 403 patients, in the period from May 1998 to December 2000. The same study was updated in 2005 (RIPID-2), with the participation of 79 medical centers in 20 regions of Italy, and has led to record 1063 patients with sarcoidosis [6, 7]. The number of identified cases, however, underestimates the real number since not all-Italian research centers have joined in the RIPID projects. Nevertheless, the Italian register presented a larger number of patients compared to that of other registers of European regions (Spain, Germany and Flanders) [7]. Despite the undeniable usefulness of this database, there are no up-to-date data in the literature and epidemiological data concerning the prevalence of patients with sarcoidosis in Italy or in specific Italian regions.

As sarcoidosis most commonly involves lungs, skin and eyes, the search for environmental and occupational causes has focused on exposure to airborne antigens. Sarcoidosis has been associated with exposure to various irritants in rural settings including wood-burning stove emissions, tree pollen, inorganic particles, microbial bio-aerosols, insecticides and mould [8–10]. A higher incidence of sarcoidosis was found in regions of Switzerland with metal industry and intense agriculture [10]. Furthermore, occupational studies of patients with sarcoidosis have shown associations with service in the U.S. Navy [11], metalworking [8], fire-fighting [12], and the handling of building supplies [9]. An increased incidence among New York City (NY, USA) Fire Department rescue workers after the 9/11 World Trade Center Attack has also been demonstrated [13–17]. The higher incidence of sarcoidosis in these workers may be due to exposure, by inhalation, to particulate matter, fibers (asbestos), organic pollution, heavy metals and fumes. Metals with antigenic properties such as barium, beryllium, cobalt, copper, gold, rare earth metals, aluminum and zirconium can promote granuloma formation [14]. Airborne antigen exposure can result from both anthropogenic and natural causes. For instance, important sources of airborne exposure are asbestos minerals and asbestiform mineral fibers [18, 19], that may induce pleural mesothelioma and interstitial lung disease. The exposition to mineral fibers can be occupational (present at the workplace) or environmental (pollution from asbestos being mined or industrially processed in the neighborhood of residence). However, in addition to these types of exposure, there are areas of the world where exposure is due to “naturally occurring asbestos”, because the mineral is a natural component of soil and rocks. Ophiolites, which are sections of the earth’s oceanic crust and underlying upper mantle that were uplifted and exposed above sea level onto continental crustal rocks by geological processes, are metamorphic rocks rich in fibrous minerals [20, 21]. Several studies have suggested a causal relationship between this last type of exposition and mesothelioma or lung disease in several rural areas of different Mediterranean regions, including Corsica, Cyprus, Greece, Italy and Turkey; and in other sites of the world, including California, Canada and New Caledonia, China [21–23].

The detection of airborne pollutants, or atmospheric monitoring, is one of the most difficult tasks in the field of environmental protection and human health. Despite stringent regulations to limit the concentration of the pollutants in soil, the amount of substances emitted into the atmosphere is still generally very high. Consequently, the air quality may be severely impaired in some areas.

The presence of particulate matter in the atmosphere, and in particular the presence of asbestiform materials and metals, can be evaluated through the analysis of the accumulation of pollutants in the tissue of mosses or lichens (organisms useful for the monitoring of air pollution).

Due to their resistance to heavy metals and to their metabolism, that is strictly dependent on atmospheric exchanges, mosses and lichens have been successfully used for decades to monitor trace elements deposition. Measuring the accumulation of airborne pollutants in these organisms affords to assess spatial and temporal deposition patterns [24–30].

Our hypothesis is that airborne antigens could play a role in the development of sarcoidosis. The goal of this study is that of determining the prevalence of sarcoidosis and distribution of cases in an Italian Province (Parma), subjected to different sources of environmental pollution, and identifying potential environmental exposures associated with sarcoidosis. We seek to determine the presence of environmental pollutants by examining the spatial clustering of sarcoidosis cases in the greater Parma area and by exploring the bioaccumulation levels of trace metals in lichen (*Xanthoria parietina* (L.) Th. F.) tissues collected in different sites of the territory.

## Material and methods

### Area of study and pollution

**Fig 1:**
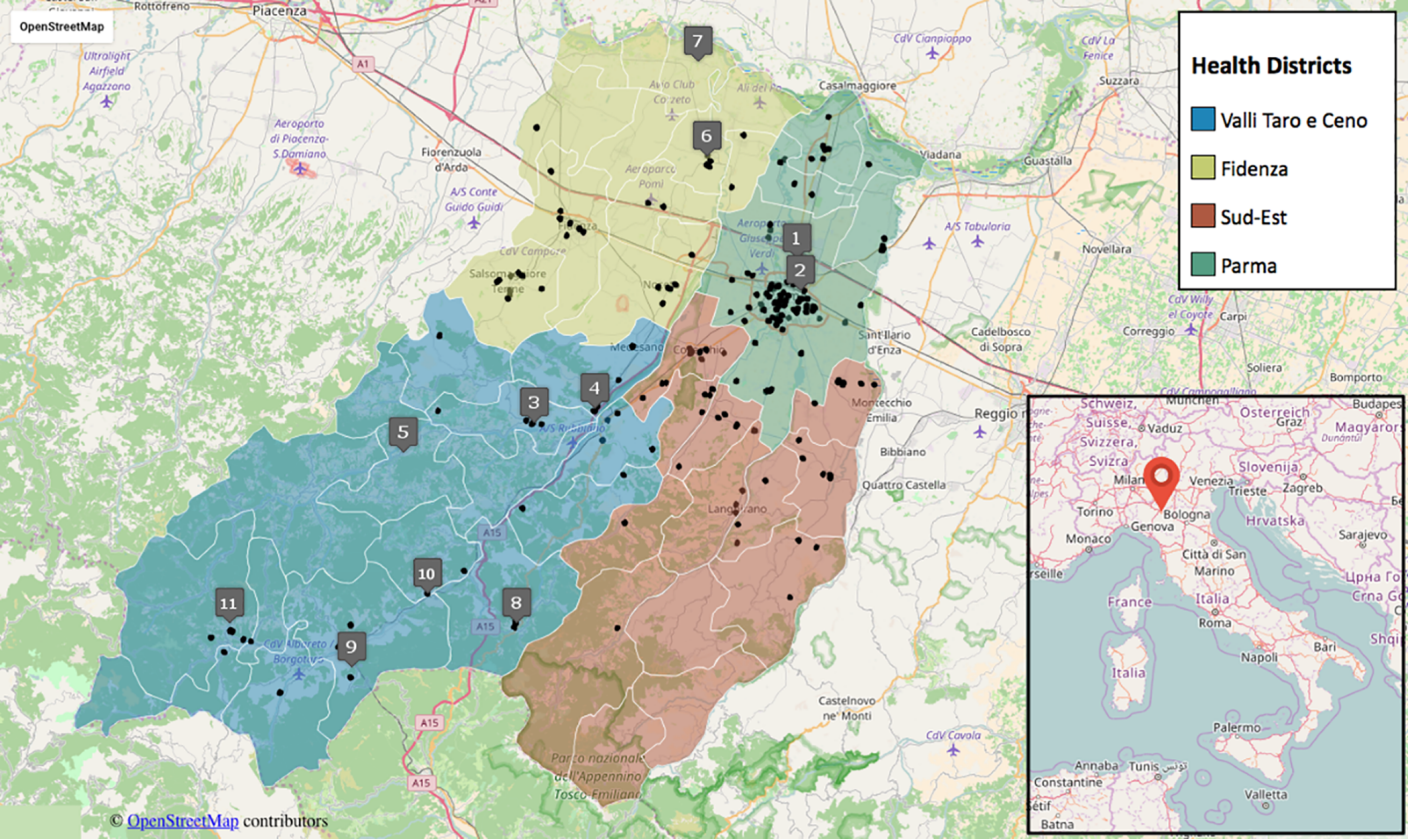
Study area, residential localization of sarcoidosis patients and location of the sampling stations, numbered as in Table 1. The inset shows the location of Parma in the Italian peninsula. Data from OpenStreetMap visualisation.

The greater Parma area (3447.4 km^2^) is comprised of 47 Municipal Districts (MDs) belonging to four Health Districts (HDs). The Parma province is located in Emilia-Romagna, a northern Italian region, characterized by the presence of a plain (Po river valley) to the north and the Apennine Mountains to the south (S1 Table and Fig 1). The areas under study are characterized by variations in elevation (from 27 meters above sea level [ASL] to 835 meters ASL) and a small climatic diversity. There are mild variation of rainfall levels and temperatures moving from the flatland to the mountain area. Data from the Regional Environmental Protection Agency (www.arpae.it) show that the annual average rainfall in the last 20 years varies between 692 and 1717 millimeters, while the annual average temperatures range from 8.0 to 14.6 degree Celsius (°C). The morphology of the soil of the greater Parma area is mostly characterized by alluvial and glacio-fluvial deposits in the flatland, marly arenaceous flysch in the hilly area and sandstone and limestone in the Apennine mountains (source Italian National Center for Soil Mapping http://www.soilmaps.it/).

The population of the greater Parma area is approximately 431.000. Population density is highest in the city of Parma, whereas the mountain areas are scarcely populated. Population data was obtained from the Italian National Institute of Statistics [31]. The province of Parma is rich of industrial settlements of manufacturing nature (food industry, metallurgic and steel industries, production of metal, wood, plastic and refined petroleum products) and construction companies (source ISTAT—NACE 2007 data frame) [31]. Due to high population density, industrialization, and poor ventilation of the Po valley, the area experienced a marked increase in air pollution since the 1960s. Pollution affects air quality not only in the large towns and industrial areas but in the whole Po valley. In fact, this area has the highest levels of air pollution in Europe [32–35]. In addition, unlike other large European lowlands, the Po valley is almost entirely cultivated. The Apennines are a collisional mountain belt that was formed during rifting and continental drift and are comprised of ophiolites [36]. These mountains are often excavated for material extraction (used in road and wall construction) and the release of fibrous material into the air during excavation may represent an environmental health hazard.

### Data collection and spatial distribution of sarcoidosis cases

Inpatients and outpatients at the University Hospital of Parma diagnosed with sarcoidosis, between 2000 and 2013, were identified for this study (S1 File). All the subjects were resident in the Parma area at the moment of diagnosis, that was performed at the University-Hospital of Parma, the only one specialized center for interstitial lung disease in the Parma province. The diagnosis is established in the presence of compatible clinical and radiological findings, histological demonstration of non-caseating granulomas, and exclusion of other known causes of granulomatous inflammations. For all patients an accurate working anamnesis was performed to collect information about many sources of possible occupational exposure (for example exposure to beryllium). Demographic data (age, gender and address) were obtained from hospital admission and discharge records or from the patients’ medical record. A total of 223 patients were identified and their demographic information was used for this study. The study was approved by the University of Parma’s Ethics Committee (protocol number 26037/2008) and was conducted in conformity with the Declaration of Helsinki.

We retrieved the geographic coordinates of residence at the moment of sarcoidosis diagnosis of each single patient and located them on the Parma Province area by a global position system (GPS). However, it is important to underline that the current residence may not reflect the onset of the disease if patients have lived elsewhere before diagnosis.

### Clinical data analyses

From the collection of clinical data obtained, the frequency of sarcoidosis in the entire province was estimated, by computing the period prevalence over the period under study (2000–2013). In order to identify possible zonal differences in prevalence, we also specialized the analysis by computing period prevalence rates based on the area of residence of patients. Rates were obtained by subdividing the population with respect to the 47 MDs, to the 4 HDs and to the altitude of the residential sites (plain, from 0 to <200 meters ASL, hill from 200 to <600 meters ASL, mountains above 600 meters ASL). For the most populated area corresponding to the municipality of Parma, prevalence was also computed inside the 13 quarters.

Statistical analysis of the prevalence dataset (see S1 Text) was performed by R package program [37], in order to determine statistically significant differences between groups (both in terms of health districts and in terms of altitude). Standard analysis of variance (ANOVA) and non-parametric (Kruskal-Wallis and Mann-Whitney-Wilcoxon,) tests (with significance level for p-values <0.05), were used.

### Enviromental biomonitoring

Eleven biomonitoring stations were chosen in the Parma province for lichen sampling after patients georeferentiation and prevalence study (Table 1 and Fig 1).

**Table 1:** Locations of the studied sites (stations) for enviromental biomonitoring.

Sites	Station name	Location description	Latitude	Longitude
1	Paradigna (Parma)	Urban area (near an industrial area); high prevalence of sarcoidosis.	44°49'56.35"N	10°20'25.67"E
2	Parma-Parco (Parma)	Urban area; absence of cases of sarcoidosis.	44°48'13.71"N	10°20'41.94"E
3	Varano de’ Melegari	Residential area in a hilly zone; high prevalence of sarcoidosis.	44°41'13.23"N	10° 0'46.43"E
4	Medesano	Residential area in a hilly zone; high prevalence of sarcoidosis.	44°41'59.38"N	10° 5'16.99"E
5	Varsi	Rural area in a hill zone; absence of cases of sarcoidosis.	44°39'40.71"N	9°50'55.83"E
6	San Secondo Parmense	Residential area in a plain zone; high prevalence of sarcoidosis.	44°55'21.93"N	10°13'43.17"E
7	Roccabianca	Residential area in a plain zone; absence of cases of sarcoidosis.	45° 0'24.21"N	10°12'59.79"E
8	Berceto	Residential area, in a mountain zone; high prevalence of sarcoidosis.	44°30'33.23"N	9°59'26.78"E
9	Borgo Val di Taro	Rural areas in a hill zone (near an ophiolites quarry); high prevalence of sarcoidosis.	44°28'12.42"N	9°47'6.56"E
10	Valmozzola	Rural area in a hill zone (near an ophiolites quarry); high prevalence of sarcoidosis.	44°32'8.43"N	9°52'46.69"E
11	Bedonia	Residential area, in a hill zone; high prevalence of sarcoidosis.	44°30'32.35"N	9°37'58.43"E

For each station, the sample analyzed was a mixture of at least 10 thalluses collected from at least three different trees. All samples were collected from *X*. *parietina* cushions within a period of 20 days during the summer of 2014. The sampling of thalluses was conducted according to the methodology described in Nimis and Bargagli [38]. Lichen specimens were collected by means of stainless steel blade with subsequent storage in Petri dishes that were sealed prior to laboratory analysis.

### Chemical data analyses

Approximately 400 milligrams (mg) of homogenized lichen tissue was treated and analyzed for trace metals as detailed in Nimis and Bargagli [38]. The material was wet digested in 65% nitric acid after adding 30% H_2_O_2_ and the concentrations of 12 elements (Al, As, Cd, Cr, Cu, Fe, Hg, Mn, Ni, Pb, Se, Zn) were determined in the digested solution (EN 13805:2014 Foodstuffs—Determination of trace elements—Pressure digestion). The determination of As, Cd, Cu, Hg, Ni, Pb, Se, Zn were been carried out by Quadrupole Inductively Coupled Plasma-Mass Spectrometry (Q-ICP-MS), (Elan 6000, Perkin Elmer Italia S.P.A., Milan, Italy). As in Q-ICP-MS determination interferences associated with spectral overlapping from concomitant isotopes or molecular ions and sample matrix composition have been identified (EN 15763–2009 foodstuffs—determination of trace elements—determination of arsenic, cadmium, mercury and lead in foodstuffs by inductively coupled plasma mass spectrometry icp-ms after pressure digestion), Inductively Coupled Plasma-Atomic Emission Spectrometry (ICP-AES) technique (Optima 5300 DV, Perkin Elmer Italia S.P.A., Milan, Italy) has been used for the determination of Al, Cr, Fe and Mn.

Both analytical methods were validated in compliance with UNI CEI EN ISO/IEC 17025 [39]. Certified Reference Materials (CRMs) BCR CRM 422 (European Commission Centre—Institute for Reference Materials and Measurements) was used to check the accuracy of analytical measurements for Al, As, Cd, Cr, Cu, Hg, Ni, Pb, Zn (Application Note 1 European Communities, Comparison of a measurement European Commission—Joint Research Centre Institute for Reference Materials and Measurements (IRMM) www.erm-crm.org). To verify the quality of the analytical data for Fe, Se and Mn fortified matrices were used. The lichen analyses were conducted at the national reference laboratory for heavy metals in inorganic matrices at the Italian National Institute of Health (ISS—Istituto Superiore di Sanità).

All data collected were subjected to statistical analysis. For each element, the values of the concentrations in the different stations were normalized by standardization. The analysis of the correlation between the metals and between stations was performed from the normalized data computing the Pearson correlation coefficient. Hierarchical clustering was applied to metal concentrations by using a distance based on Pearson correlation coefficients between stations, then performing identification of groups using the complete linkage criterion. The resulting classification was displayed in the form of a dendrogram. Principal component analysis (PCA) has been used as an alternative way to represent inter-stations relationships. All statistical analyses were performed using R software [37].

## Results

### Characteristics, prevalence and spatial distribution of sarcoidosis patients

A total of 223 patients were identified from 2000 to 2013, among them 130 female and 93 male patients (58.3% and 41.7% of total, respectively). About 93% of the ill population was of Caucasian ethnicity whereas 7% was of other ethnic groups. The average age of the patients identified for this study was 50.6 years. Table 2 shows in detail the demographic characteristics of patients and of background population of Parma province.

**Table 2:** Characteristics of sarcoidosis patients and of general population in Parma province.

Characteristics	All patients	Characteristics	All population
Number	223	Number	447251
Gender (%)		Gender (%)	
Female	130 (58.3)	Female	229760 (51.37)
Male	93 (41.7)	Male	217491 (48.63)
Age at diagnosis (mean)	50.6 ± 15.4	Age (mean) 2013	44.4
Female	53.5 ± 15.5	Female	46.4
Male	46.5 ± 14.4	Male	42.9
Frequency for ranges of age (%) in year 2013		Frequency for ranges of age (%) in year 2013	
<30	6.1	<30	9.1
30–39	23.5	30–39	11.2
40–49	22.5	40–49	16.7
50–59	16.3	50–59	16.4
60–69	16.8	60–69	16.2
>69	14.8	>69	30.4
Ethnicity (N, %)		Ethnicity (N, %)	
Caucasian	207 (92.8)	Caucasian	386701 (86.5)
Other	16 (7.2)	Other	60550 (13.5)

**Fig 2:**
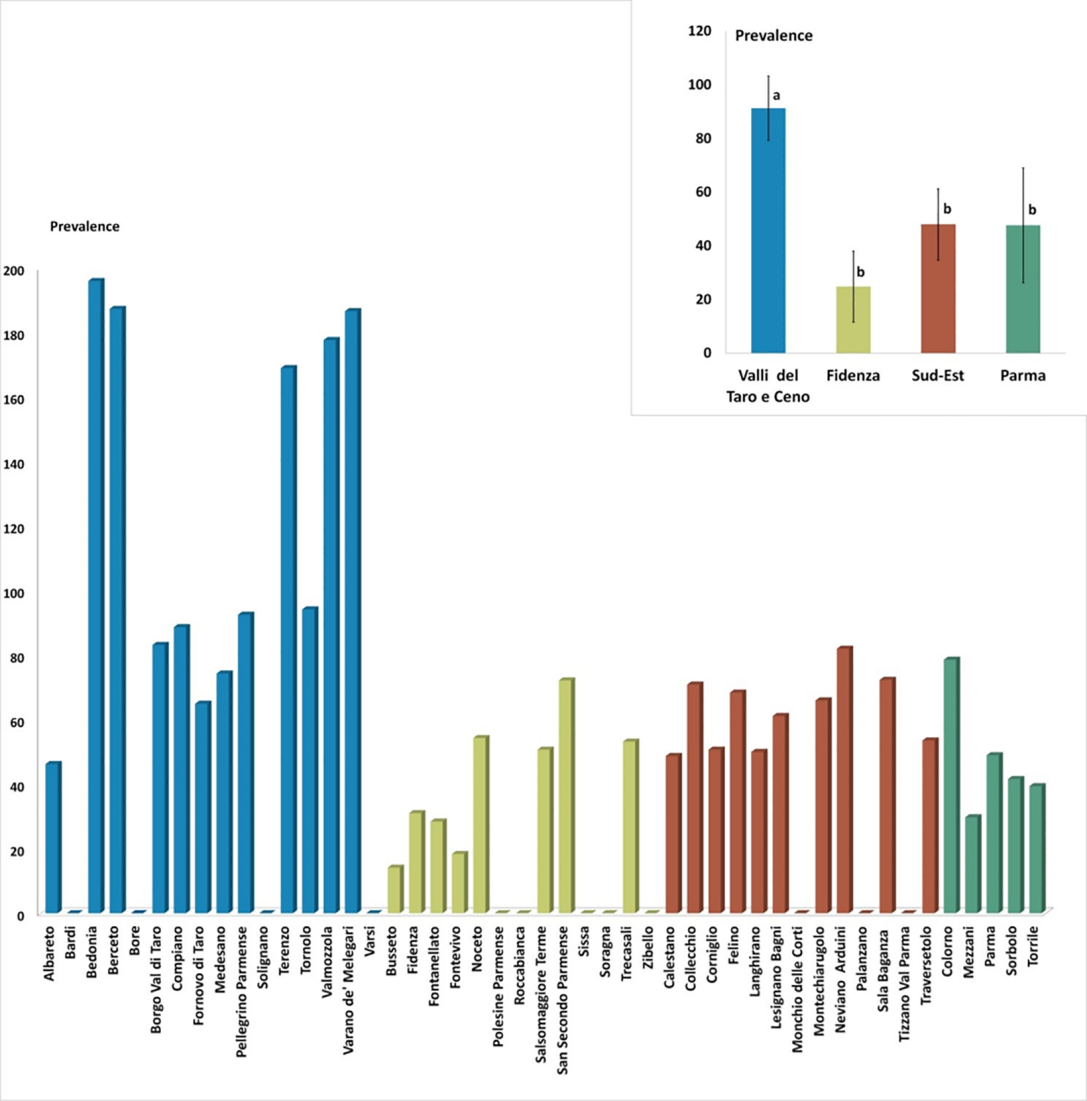
Graphic representation of the sarcoidosis prevalence for municipality (MDs) and health districts (HDs). In the upper right, graphic representation of the sarcoidosis mean prevalence health districts groups compared using ANOVA to Fisher (Significance is expressed with different letters [a, b] and by level of significativity expressed by p values <0.005).

We observed that the prevalence in the province of Parma over the period 2000–2013 was of 49 cases per 100.000 individuals. The MD with the highest prevalence was “Bedonia” (prevalence of 196 per 100.000) followed by “Berceto” and “Varano de’ Melegari” (187 per 100.000), “Valmozzola” (178 per 100.000) and “Terenzo” (169 per 100.000) MDs. All these MDs with a prevalence higher than 100 per 100.000 belong to “Valli Taro e Ceno” HD. For 19 MDs, the prevalence was between 50 and 99 per 100.000, whereas 11 MDs showed a prevalence less than 50 per 100.000. The following MDs did not present any case of sarcoidosis: “Bardi”, “Bore”, “Solignano” and “Varsi” (“Valli Taro e Ceno” HD), “Polesine”, “Roccabianca”, Sissa”, “Soragna” and “Zibello” (“Fidenza” HD), “Monchio delle Corti”, “Palanzano” and “Tizzano Val Parma” (“Sud-Est” HD) (Fig 2, S1 Fig and S2 Table). When considering subsets of MDs in the province, we adopted two different measures of period prevalence: we call “mean period prevalence” over a group of municipalities the empirical mean of the period prevalence values of the MDs forming the group; we address instead as “aggregate period prevalence” (APP) the period prevalence computed by considering the aggregate number of cases and the aggregate population in the group area. When the two quantities are similar for a group of MDs under study it means that there is no much internal heterogeneity. The mean period prevalence was about 91 per 100.000 individuals in the “Valli Taro e Ceno” HD (aggregate period prevalence equal to 89), about 48 in “Parma” (APP 49) and “Sud-Est” (APP 60) HDs, and about 25 in “Fidenza” HD (APP 34).

By grouping the MDs according to the altitude, we observed that in the towns and villages of the lowland (below 200 meters ASL) the mean period prevalence was about 47 per 100.000 individuals (aggregate period prevalence equal to 49), in the hilly areas (between 200 and 600 meters ASL) it was about 84 (APP 76) while in the mountain areas (over 600 meters ASL) it was approximately 42 (APP 48).

The result of one-way ANOVA test reported in Fig 2 suggests that the “Valli Taro e Ceno” HD presented a significantly higher prevalence compared to the other districts (p-value < 0.05). The result of the parametric analysis is confirmed by non-parametric tests discussed in the S1 Text. Similarly, when grouping MDs as function of the altitude, hilly areas displayed a moderately larger prevalence than the rest of the dataset (see S1 Text).

**Fig 3:**
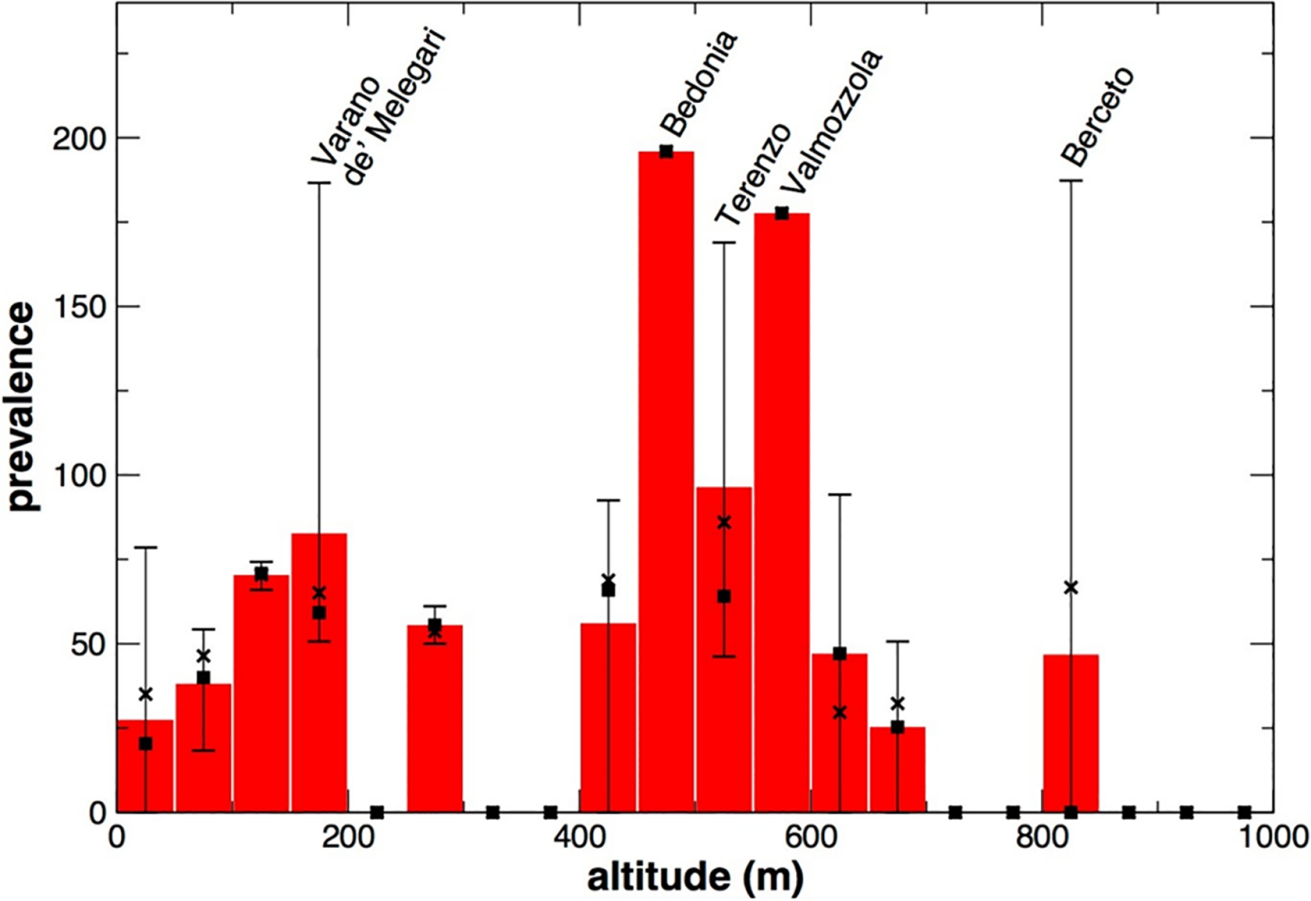
Histogram representing the mean prevalence of sarcoidosis as function of the altitude, obtained grouping together MDs over intervals of 50 meters of altitude. The highest and lowest values of prevalence in the municipalities belonging to each interval are indicated by the bars; a square symbol identifies the median value in each group. The values of the aggregate prevalence for each group are indicated by crosses.

A more detailed analysis of the distribution of the disease depending on the altitude is shown in Fig 3, where the statistics of prevalence is computed over groups of MDs within intervals of 50 meters of altitude. The mean period prevalence resulted larger at altitudes between 450 and 600 meters above sea level; the same occurred for aggregate period prevalences (Fig 3).

**Fig 4:**
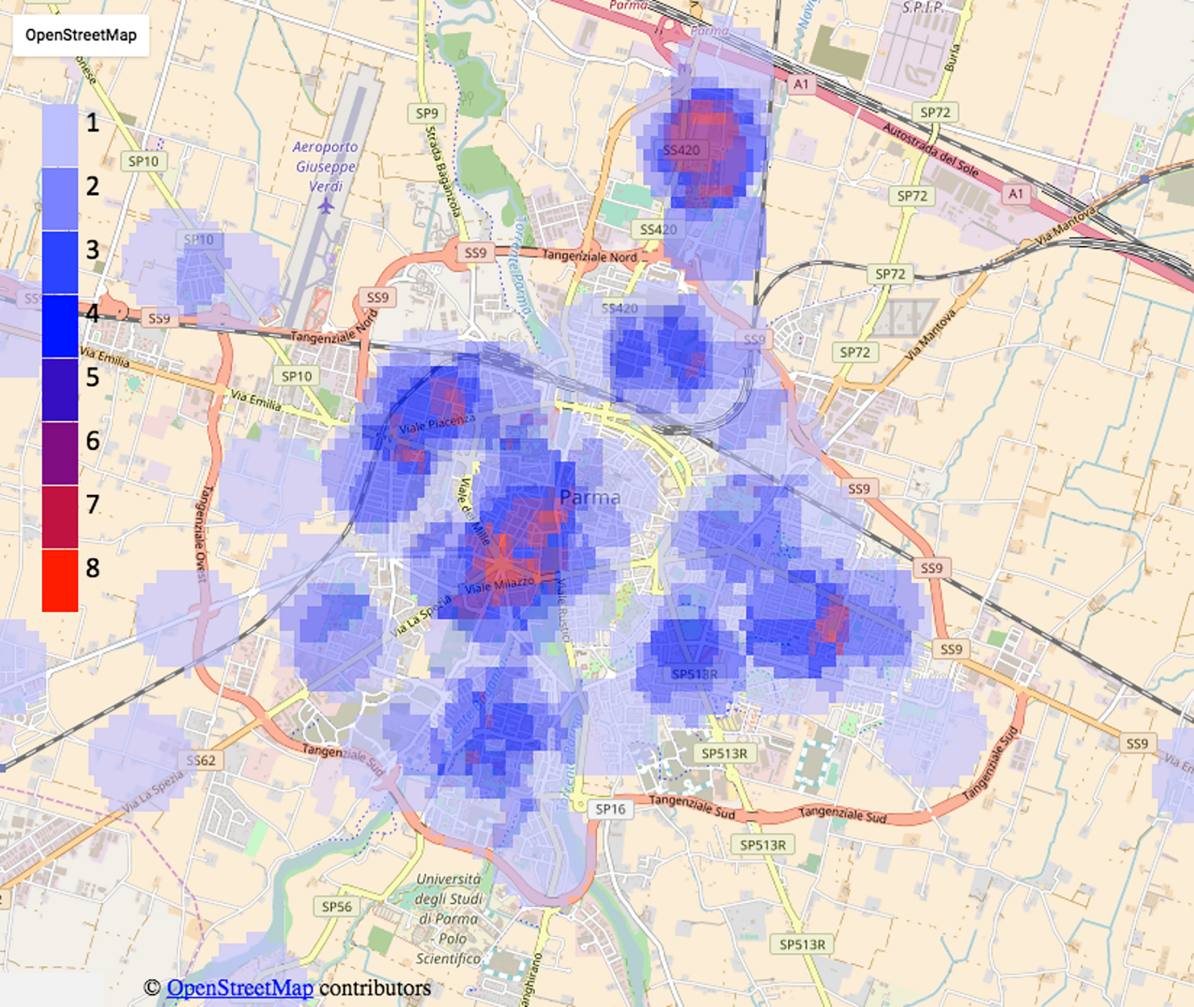
Heat map of distribution of sarcoidosis cases the Parma area. The color-scale from low blue (equal to 1) to red (equal to 8) displays the number of patients resident within a range of 500 meters from each considered point on the map of the area under study. Data from OpenStreetMap visualisation.

With the information obtained for each patient, we identified their coordinates of residence and evaluated the spatial segregation of the disease in Parma province (Fig 1). By means of the georeferentiation of patients, it was possible to observe some groupings (clusters) of cases of sarcoidosis throughout the province. Looking at the microscopic level, we found that, in both towns and villages, and regardless of the altitude, the residences of some patients were aggregated, that is they were in a close neighborhood or even in the same building (e.g. we identified 5 buildings with 2 patients each, data not shown). In particular, analyzing patients only within the town of Parma, we found at least two critical areas in which the number of patients was much larger compared to the neighboring areas. This is highlighted in Fig 4, where, for each point on the map, a color code (from low blue to red) indicates the number of patients within a range of 500 meters. The red colored areas on the map represent a local aggregate up to 8 patients, while the areas with no color indicate the absence of cases within 500 meters.

**Fig 5:**
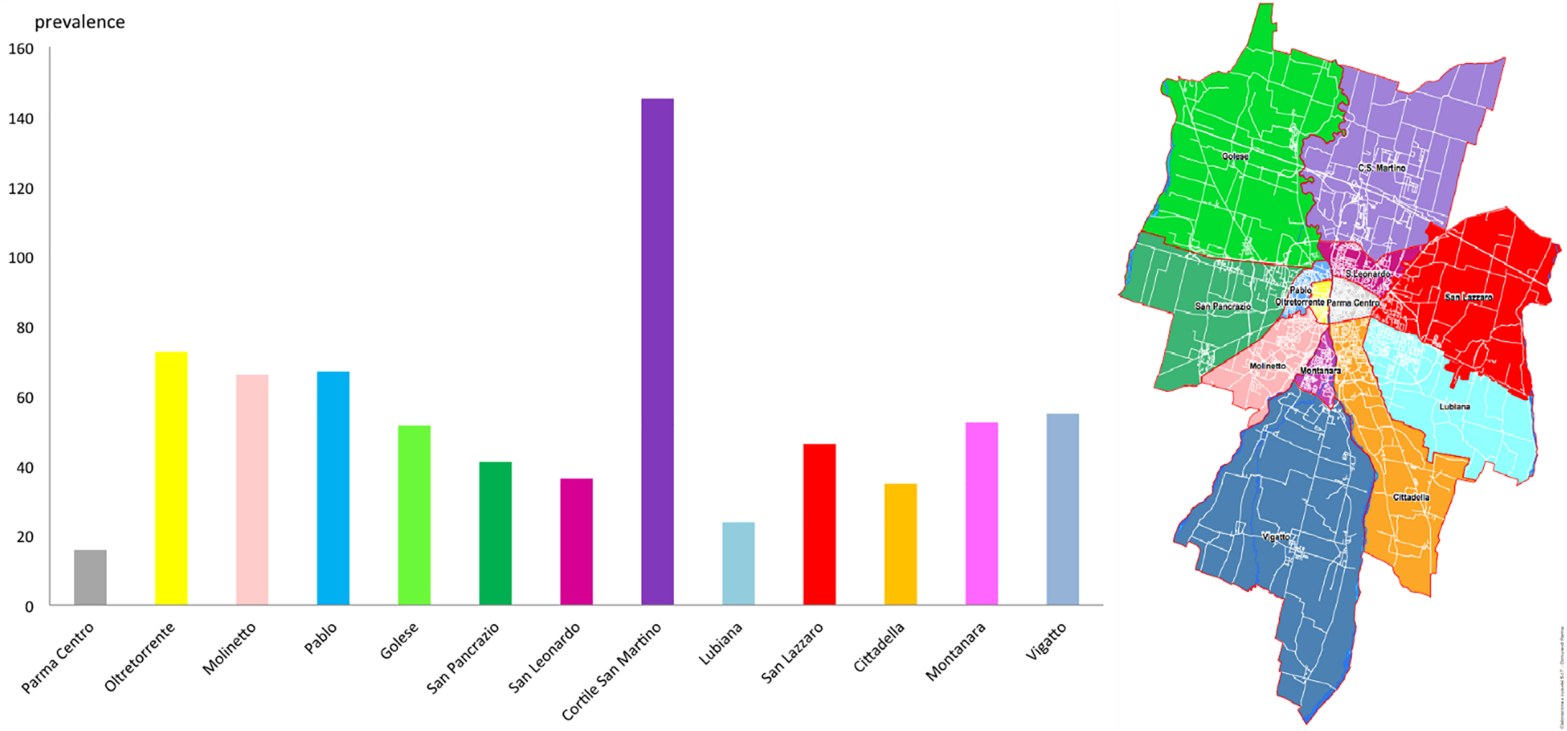
Histogram of the prevalence in the quarters of the Parma town. The inset shows the location of quarters in the town.

These results are confirmed by the analysis of the prevalence obtained subdividing the population with respect to the 13 Quarters of Parma district (Fig 5). Fig 5 shows that the Quarter “Cortile San Martino”, which includes the first cluster of patients (the red cluster in the northern area visible in Fig 4, and close to the “Paradigna” monitoring station), presented a significantly higher prevalence of patients compared to all other Quarters of Parma. The second major cluster (close to the center of the town), instead, is not highlighted by the analysis of prevalence because it is located at the junction of 3 different Quarters (“Molinetto”, “Oltretorrente” and “Pablo”).

### Enviromental biomonitoring

Table 3 and S2 File report the values of trace elements found in the samples of *X*. *parietina*. Aluminum is constantly the most abundant element in all the stations under study, assuming variable values between about 250 and 700 mg kg^-1^ (dry weight). Other elements (Fe, Zn, Cu, Pb, Ni), which have both anthropogenic and terrigenous origins, presented values ranging between about 1 and 50 mg kg^-1^ (dry weight); As, Cd, Hg and Se had concentrations below 1 mg kg^-1^(dry weight), whereas it was not been possible to quantify Cr and Mn because their concentrations were lower to the detection limit of the instrument (ICP-AES) in all samples.

**Table 3:** Concentrations of trace metals in *X*. *parietina* tissues from 11 sites in the Parma province and prevalence of sarcoidosis MDs (the prevalence sites labeled with an asterisk refer to Parma MD).

Site	Prevalence	Trace metals (mg Kg^-1^ dry wt.)											
		As	Al	Cd	Cr	Cu	Fe	Hg	Mn	Ni	Pb	Se	Zn
Paradigna (Parma)	48.95*	0.165±0.07	417.45±53.9	0.094±0.043	n.q.±/	8.71±2.01	10.12±2.29	0.125±0.055	n.q.±/	3.41±0.907	1.518±0.456	0.209±0.0846	47.157±8.448
Parma-Parco (Parma)	48.95*	0.179±0.07	400.4±52.0	0.056±0.028	n.q.±/	11.89±2.62	10.67±2.39	0.1±0.045	n.q.±/	1.716±0.506	1.034±0.329	0.187±0.077	42.185±7.685
Varano de' Melegari	186.57	0.012±0.01	251.35±35.0	0.01±0.006	n.q.±/	2.76±0.76	5.72±1.41	0.057±0.028	n.q.±/	0.836±0.275	0.407±0.149	0.088±0.041	11.726±2.590
Medesano	74.29	0.216±0.09	524.7±65.4	0.046±0.023	n.q.±/	7.04±1.68	12.76±2.78	0.133±0.058	n.q.±/	3.938±1.025	1.474±0.445	0.088±0.041	41.195±7.531
Varsi	0	0.197±0.08	403.15±52.3	0.01±0.006	n.q.±/	4.85±1.22	9.13±2.09	0.165±0.069	n.q.±/	2.255±0.638	1.045±0.332	0.088±0.041	23.617±4.695
San Secondo Parmense	72.15	0.188±0.08	465.85±59.1	0.019±0.011	n.q.±/	7.48±1.77	11.11±2.47	0.056±0.028	n.q.±/	2.75±0.756	1.628±0.484	0.2046±0.083	45.848±8.248
Roccabianca	0	0.145±0.06	278.85±38.2	0.067±0.032	n.q.±/	5.66±1.40	6.16±1.50	0.056±0.028	n.q.±/	1.639±0.487	1.342±0.411	0.088±0.041	34.496±6.477
Berceto	187.27	0.185±0.08	536.8±66.7	0.01±0.006	n.q.±/	4.66±1.18	11.99±2.64	0.05±0.025	n.q.±/	2.31±0.652	1.925±0.558	0.088±0.041	24.277±4.805
Borgo Val di Taro	83.12	0.207±0.08	609.4±74.3	0.041±0.021	n.q.±/	3.89±1.01	11.66±2.58	0.065±0.031	n.q.±/	1.749±0.514	1.1±0.347	0.088±0.041	25.003±4.928
Valmozzola	177.62	0.111±0.05	718.3±85.4	0.01±0.006	n.q.±/	31.86±6.05	46.75±8.39	0.082±0.038	n.q.±/	7.678±1.808	2.497±0.696	0.088±0.041	36.949±6.867
Bedonia	195.91	0.093±0.04	416.9±53.8	0.01±0.006	n.q.±/	5.15±1.29	7.59±1.79	0.036±0.019	n.q.±/	1.012±0.323	1.353±0.414	0.088±0.041	18.986±3.9

The correlations analysis between the concentrations of the elements shown in S2 Fig provides a way to detect possible deposition patterns.

The elements that have the greatest number of significant associations (p value <0.05) are: Ni with Cu (r = 0.89), Fe (r = 0.92), Al (r = 0.73), Pb (r = 0.80); Fe with Cu (r = 0.95), Ni (r = 0.92), Al (r = 0.76), Pb (r = 0.75); Al with Fe (r = 0.76), Pb (r = 0.74), Ni (r = 0.73), Cu (r = 0.61); Cu with Ni (r = 0.89), Fe (r = 0.95), Al (r = 0.61) Pb (r = 0.7); Pb with Al (r = 0.74), Fe (r = 0.75), Cu (r = 0.70), Ni (r = 0.80); Zn with Se (r = 0.72) and Cd (r = 0.62); Se with Zn (r = 0.72); Cd with Zn (r = 0.62).

**Fig 6:**
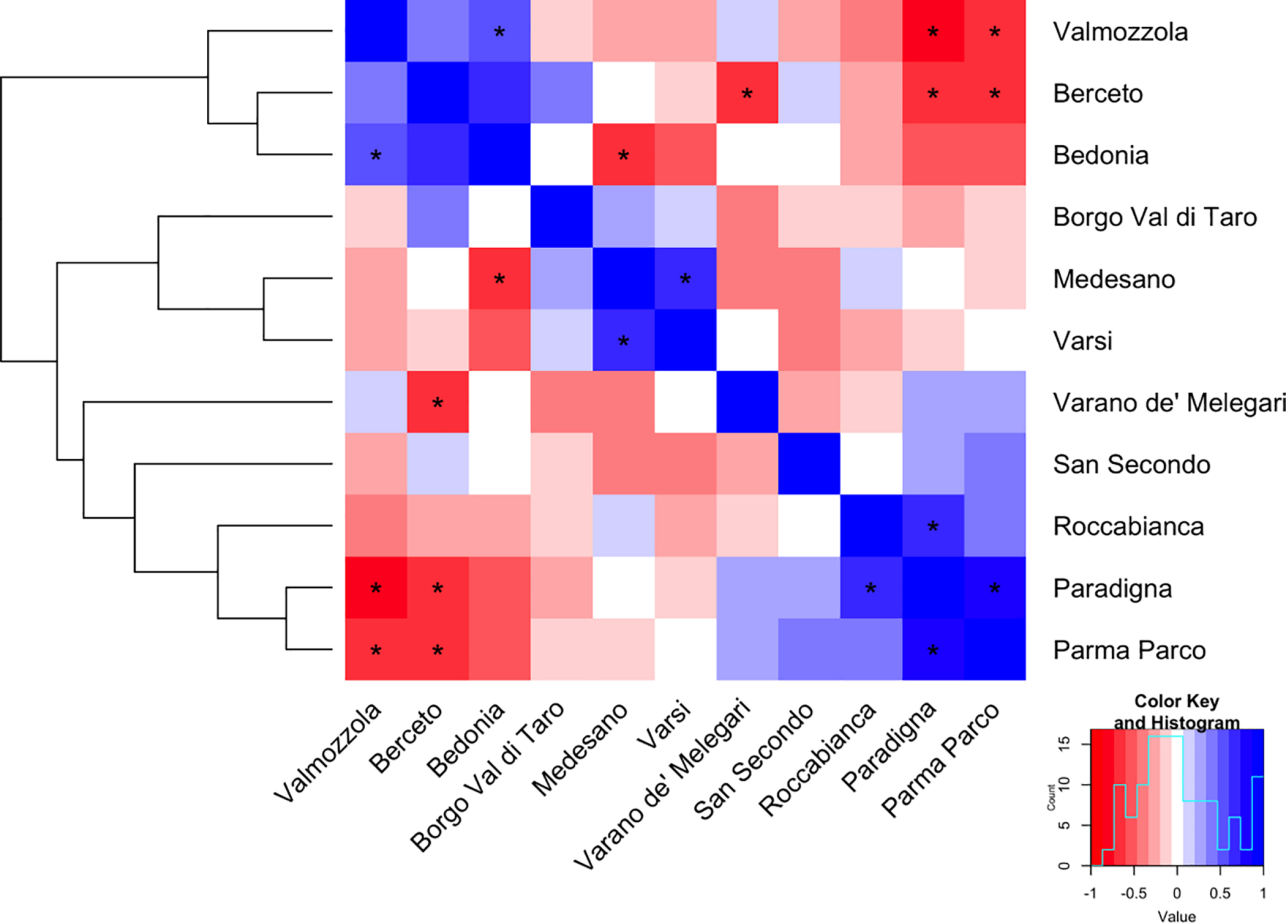
Heat-Plot representing the correlations among different stations computed from the concentrations of the metals under study and associated dendrogram obtained from the hierarchical clustering analysis of the same data computed using a distance based on Pearson correlation coefficients and complete linkage. The associations for which the value of correlation is significant (p value < 0.05) are marked with an asterisk.

Subsequently, we used the same set of data in the orthogonal way, to correlate and classify the sampling stations in relation to their exposure to particulate air pollution (metals in trace identified). The heat-map in Fig 6 uses a color scale to represent Pearson correlation coefficients between stations. Significant correlations (p value < 0.05) are labeled with an asterisk. The existence of some correlation patterns is also remarked by a dendrogram obtained performing hierarchical clustering with correlation distance and complete linkage criterion. Due to the small number of significant associations, only major overall features of the hierarchical structure, not the details, should be taken into account. In this respect, the structure of the dendrogram approximately reproduces the altitude profile of the set of stations, with stations from mountain area well separated from those of lowlands.

Similar group structures were detected with a multivariate principal component analysis (PCA), in which the first (PC1) and second (PC2) principal components explain 70.80% of the total variance among stations. A comparison of scores and loadings for F1 and F2 makes possible the identification of the metals in trace having a greater influence on the ranking of stations (S6 Fig and S3 Table). In PCA results, a first cluster composed by the stations “Paradigna”, “Parma-Parco”, “Roccabianca” and “San Secondo Parmense” is mainly characterized by the presence of Cd, Zn, Se, As, Hg, whereas, a second cluster, composed by stations from “Valli Taro e Ceno” HD, by the deposition of Fe, Al, Ni, Cu, Pb.

## Discussion

This study was aimed to identify and analyze a pool of patients with sarcoidosis resident in the province of Parma. As previously described, such area is characterized by a rather heterogeneous territory, in which different sources of environmental pollution, marked in the literature as potential antigens for the onset of sarcoidosis or other respiratory diseases are present. In this respect, it would be important to identify possible correlations between the presence/frequency of the disease and sources of environmental pollution.

We have identified a cohort of 223 patients. Most patients were of Caucasian ethnicity, as expected, since the 93.18% of the total resident population was Caucasian [31]. Sarcoidosis was more frequent among females, although the number of females is only slightly higher than the number of males in the background population. The calculated mean age of the patients at the time of diagnosis was approximately 50 years. In fact the disease is more present in young adults but, according to many studies, the first diagnosis is made in adults with a mean age of about 50 years [6, 10]. In the ACCESS (A Case-Control Study of etiologic Sarcoidosis) study the peak age was between 35 and 45 years [40], while in a UK survey was found between 35 and 55 [41]. The review of Valeyre et al. [3] indicates that 70% of patients is diagnosed between 25 and 45 years. It is important to say that the age of diagnosis very often does not coincide with the onset of the disease. This is because sarcoidosis is in many cases asymptomatic or it has similar symptoms to other diseases [42, 43]. Gentry et al. [44] have shown that in many patients the disease was discovered accidentally by routine X-ray examination taken during the course of hospitalization. Musellim et al. [43] have identified a higher average age of diagnosis for female patients compared to males, with a difference of 10 years between the two genders. In our study, we found a similar result, with an average age of diagnosis that is approximately 53 years for females and 46 years for males. We recorded a discrete number of patients older than 60 years, mainly females (37%), as found also in Scandinavian, Japanese and Spanish studies [43, 45]. This could be justified by the fact that these patients may have discovered the disease after years from its onset, because of a worsening in their state of health.

Sarcoidosis is included in the registry of rare diseases, the Orphanet Reporter Series for Rare Diseases, a resource used by the NIH’s Office of Rare Lung Diseases Research to track disease prevalence. The registry reported an estimate of the mean worldwide sarcoidosis prevalence of 12.5 cases per 100.000 individuals (www.orpha.net/orphacom/cahiers/docs/GB/Prevalence_of_rare diseases_by_alphabetical_list.pdf). In this respect, the present work revealed a much higher prevalence for the whole population under study, estimated at about 50 cases per 100.000. On the other hand, previous studies have shown that sarcoidosis has a higher prevalence compared to that of other rare diseases of the lung; it is also emphasized that sarcoidosis is much more common than predicted [46].

At the level of individual MDs the average prevalence measured in the present study revealed an interesting heterogeneity, with values ranging from 0 to about 195 per 100.000 cases, although these values should be taken with caution because of the very small number of cases observed in some MDs. The MDs with higher prevalence belong to the “Valli Taro e Ceno” HD, a wide district that, together with the “Sud-Est” HD of the province, groups all the MDs of the hill and mountain areas of the province. In line with this result, the average prevalence calculated according to the altitude of residential locations was higher in the hilly areas, suggesting that an environmental component present in these areas could be at the base of the onset of the disease.

The “Valli Taro e Ceno” HD consists of small population centers located in cultivated rural areas and wooded areas. From a geological point of view, moreover, these areas are characterized by the presence of ophiolite outcrops, already object of investigations by other research studies, as risk factors for the onset of mesothelioma [47]. In the areas surrounding the centers of some MDs with high prevalence (“Bedonia”, “Berceto”, “Valmozzola”, “Varano de' Melegari”, “Terenzo”, “Tornolo”), there are quarries at these minerals (some of which are no longer used). However, others MDs, such as “Bardi”, had no cases of sarcoidosis while being characterized by the presence of ophiolitic quarries (these have been abandoned between 2001 and 2011). An interesting result emerged when considering the geographical distribution of prevalence rates, depicted in the form of a heat-map on the map of the province (S1 Fig): The map shows that, within the “Valli Taro e Ceno” HD, a continuous region of high prevalence can be identified in the hilly and mountain areas of the High Taro river Valley (comprising high-prevalence MDs such as “Bedonia”, “Berceto”, “Valmozzola”, “Borgo Val di Taro”). On the other hand, the High Ceno river Valley is completely free of ascertained sarcoidosis cases (see MDs such as “Bore”, “Varsi”, and “Bardi”). Although the two valleys are geographically close, the environmental conditions could be considerably different as the Taro valley is characterized by several communication infrastructures (highway, railway) and larger number of industries (in “Bedonia” and “Borgo Val di Taro” MDs) and mining and quarrying sites compared to the other valley. In order to check the statistical relevance of of this observation, we performed a spatial clustering analysis as in Nicholson et al. [48] by means of the SaTScan^TM^ software, where the number of cases in each MD was used as a case file, the corresponding background population (year 2013) as a population file, while the coordinate file contained the latitude and longitude coordinates of the MDs. We considered a discrete Poisson probability model. SaTScan^TM^ software uses Kulldorf spatial scan statistic to identify clusters of circular shapes and determines the significance on the bases of multiple tests depending on the underlying population model and on the maximum allowed cluster size (up to 50% of the population). With 10% population threshold, the method was able to identify a low prevalence rate cluster in the lowland area (comprising “Polesine P.”, “Zibello”, “Busseto”, “Roccabianca”, “Soragna” and “Sissa”) with 4.4 prevalence for a total population of 22582 individuals and an estimated relative risk of 0.08 (p-value 0.0037). Searching for high-rate clusters, with 10% population threshold, the method was able to identify a large cluster of about 40km of radius comprising most MDs from the “Valli Taro e Ceno” HD (“Bedonia”, “Tornolo”, “Compiano”, “Albareto”, “Borgo Val di Taro”, “Bardi”, “Valmozzola”, “Varsi”, “Bore”, “Berceto”, “Solignano”, “Pellegrino P.”, “Corniglio”, “Varano de' Melegari”, “Terenzo”), with a population of 30902 individuals, a prevalence of 96.9 and estimated relative risk 2.01 (p-value 0.041). Decreasing the population threshold to 5%, the cluster is reduced to 5 MDs (“Solignano”, “Varano de' Melegari”, “Valmozzola”, “Terenzo”, “Berceto”) with 8339 individuals, prevalence 143.6 and estimated relative risk 2.88 (p-value 0.064).

Through the georeferentiation of the registered cases, it was possible to relate the collected data directly to the territory, allowing for a more detailed description compared to analyses based on prevalence only. Patients are not evenly distributed nor are concentrated in areas with higher population density. According to other studies [10], it is reasonable to observe fair percentages of cases in rural areas and in isolated areas, away from the residential ones. In our study, we also observed many patients of different MDs living in industrial areas or residential areas adjacent to the industrial districts. In some cases, in fact, there seems to be a preferential localization to the peripheral areas of some towns, in particular in the surrounding areas to industrial centers and rural farming zones. In particular, in the MD of Parma, we identified two clusters characterized by a high density of patients. We would like to emphasize that a larger number of patients in a given area does not correspond to a higher population density. It is interesting to remark the low prevalence found in the neighborhood of the city center of Parma, which is densely populated but also characterized by pedestrian or traffic-limited areas and by the absence of industrial activities. The first cluster (Paradigna) is characterized by a housing estate, adjacent to a heavily industrialized area (factories of glass and ceramic materials, metal processing, drugs productions), to heavy-traffic roads, among which a highway, and, but only recently, an incinerator. For the second cluster, which is located in a residential area adjacent to the city center, we have not identified potential sources of pollution apart from heavy urban traffic. These observations are confirmed by the analysis of the prevalence observed in the Quarters of MD Parma (Fig 5).

Unfortunately in the all province we do not have information on the exact location of the industries, therefore any more detailed investigation of possible residential exposures due to environmental industrial pollution is beyond our possibilities.

The environmental biomonitoring through the analysis of trace metals accumulated in *X*. *parietina* was an effective method to identify the presence of mineral particles, including intake of terrigenous elements and derived from industrial complexes, that are possible antigens for sarcoidosis and other respiratory diseases. Moreover, the accumulation of trace elements has reflected atmospheric deposition levels in the different areas under sampling and the results have represented a snapshot of the environmental gradient that is visible in the province. This is highlighted by the analysis of correlations between pickup stations and between trace elements.

The correlation and classification of the sampling stations in relation to their exposure to particulate air pollution (metals in trace identified), in Fig 6, well represent the altitude profile across Parma province, with stations from the mountain area well separated from lowland stations.

Moreover, the different clusters are characterized by rather different prevalence levels: 191 for the first cluster (mountain areas) and 52 for the second, lowland and hilly stations, which in turn is formed by two clusters of prevalence 72 and 51). Computing the 95% confidence intervals for the prevalence values of the two clusters (98–334 and 43–64), we can confirm a statistically relevant difference between them.

The lowland area is characterized, in general, by the presence of anthropogenic elements, both derived from industrial activities and from rural/agricultural activities. In fact, the principal component analysis (S6 Fig) identified a cluster of lowland stations (Paradigna, Paradigna-Parco, Roccabianca, San Secondo Parmense) with positive correlation among elements Cd, Zn, Se, As, Hg. In this respect, we remark that "Paradigna" station is located close to the major industrial suburban area of the city of Parma, hence it is reasonable to assume that the contribution of these elements depends on the short-range transport of heavy metals of anthropogenic origin.

The second cluster (stations in the “Valli Taro e Ceno” HD) is characterized by highly correlated concentration values of some metals (Fe, Al, Ni, Cu, Pb) that can be associated with a contamination of twofold nature: the contribution of ophiolitic substrate and an anthropogenic pollution (industrial activity). For example, Fe and Al are used in the chromium-plating plants, in the production of paints, in mechanical industry in general, while nickel is employed in the ceramic industries. Fe, Al are also elements mainly present in the soil and together with the Ni are present in ophiolitic materials [27, 49]. Moreover, the high correlation individued between Al, Fe, Ni, Pb are in line with other studies [27]. Among the stations of “Valli Taro e Ceno” HD, Valmozzola deserves a special remark, because it presented the highest deposition values of all the elements analyzed with respect to all other stations. A possible justification of the high levels detected is that the Valmozzola MD is characterized by the presence of an ophiolite quarry, a railway station and a highway. The coexistence of these sources of contamination could be the explanation for the higher prevalence of sarcoidosis in some areas of “Valli Taro e Ceno” HD.

These observations are in accord with epidemiological data that support the hypothesis that environmental, occupational and paraoccupational agents play an important role in the development of sarcoidosis [50]. It has been well established that a number of metals can cause granulomatous inflammation as sarcoidosis and disease that mimics sarcoidosis, including beryllium, zirconium, titanium, nickel, chromium, cobalt, silicon, earth elements, and aluminum [8] [50–52]. Moreover, other studies report that man-made mineral fiber, hard rock dust (silica) and metal dust and silicates possess antigenic properties which promote granuloma formation [51].

This study has some limitations. First, at the level of municipalities, the prevalence rates cannot be considered as stable, and therefore completely reliable, because of the small numbers involved, both in terms of cases and population sizes. For this reason, our findings must be limited to our Italian province: a study involving large-cohort of cases in a large population is then need. Second, the study has not provided an analysis about the presence of beryllium in the pool of metals evaluated. Although in general sarcoidosis and chronic beryllium disease are clinically indistinguishable, an accurate working anamnesis was performed in all patients enrolled to identify possible occupational exposure to beryllium. Third, although the deposition and the consequent bioaccumulation of airborne elements is influenced by several conditions, such as geographical variations, precipitations, air mass and soil dust, the sampling method adopted in the present research allowed to take samples as much as possible homogeneous and comparable with each other while minimizing the variability in the data. Moreover, as described above it is necessary to extend the analysis to the whole great province in study, investigating other elements not studied in this work (e.g. Be, Co, Si,).

## Conclusion

In conclusion, this study is the first attempt to produce a record that would collect all cases of sarcoidosis from 2000 to 2013 in the province of Parma. Several areas of high prevalence were found, with values considerably larger than those reported in literature. A significantly higher mean prevalence was found for MDs belonging to the “Valli Taro and Ceno” HD and hilly areas in general. A notable result obtained from georeferentiation of the reported cases is the individuation of a phenomenon of aggregation of cases that was qualitatively observed at the province level and quantitatively demonstrated for the municipality of Parma.

Although the high prevalence found in some areas, the number of actual clinical cases examinated and monitored as well as the number of selected pickup stations and metal samples considered in this preliminary study are not sufficient to establish a clear correlation between the onset of sarcoidosis and environmental risk factors. These results suggest a more in depth study of the etiology of the disease, especially in areas with high number of sarcoidosis cases.

## Supporting information

S1 Fig Heat-map representing the prevalence rates of sarcoidosis for the 47 MDs in the area under study (the province of Parma). The red color scale indicates the prevalence level from zero (no cases) to 200/100.000 individuals (see S2 Table). Data from OpenStreetMap visualisation.
(TIF)

S2 Fig Correlation Matrix of the trace elements in *X*. *parietina*. All values of Pearson correlation coefficient between elements are reported. Significant correlations are highlighted by circles (p-value <0.05).
(TIF)

S3 Fig Boxplot representing the distribution of prevalence data in the four Health Districts under study. Full line shows the median value of the distirbution, lower and upper hinges correspond to the first and third quartile respectively, whiskers extend to the 95% configence interval, while data beyond that are plotted with points (outilers).
(TIF)

S4 Fig Quantile vs. quantile plot for the prevalence data (points) compared with a fit by a normal distribution (red line). Departures from the line indicate that data are not normally distributed.
(TIF)

S5 Fig Boxplot representing the distribution of prevalence data in the three altitude-based groups under study. Full line shows the median value of the distirbution, lower and upper hinges correspond to the first and third quartile respectively, whiskers extend to the 95% configence interval, while data beyond that are plotted with points (outilers).
(TIF)

S6 Fig PCA plot of ten element in trace detected in *X*. *parietina* for eleven stations of Parma province. (TIF)

S1 Text Statistical Analysis of the prevalence data (Health Districts and altitude-based groups). (DOCX)

S1 Table Characteristics of Municipalities Districts (MDs) belonging to four Health Districts (HDs) of Parma province. (DOCX)

S2 Table Number of cases and prevalence of sarcoidosis in Municipalities Districts (MDs) of the Province of Parma to Health Districts (HDs) (2000–2013). (DOCX)

S3 Table Variable Contribution (%) to the Principal Components (F1 and F2). (DOCX)

S1 File List of patients according to the MD and HD. (PDF)

S2 File Concentrations of trace metals. (PDF)

## Abbreviations

GPS: Global position system
HDs: Health districts
ICP-AES: Inductively coupled plasma—atomic emission spectrometry
MDs: Municipal districts
Q-ICP-MS: Quadrupole inductively coupled plasma—mass spectrometry
