# Supplementary figures and images for: Sarcoidosis in an Italian province. Prevalence and environmental risk factors
Source: PLoS One. 2017 May 5;12(5):e0176859. doi: 10.1371/journal.pone.0176859 (PMC5419555; doi:10.1371/journal.pone.0176859)

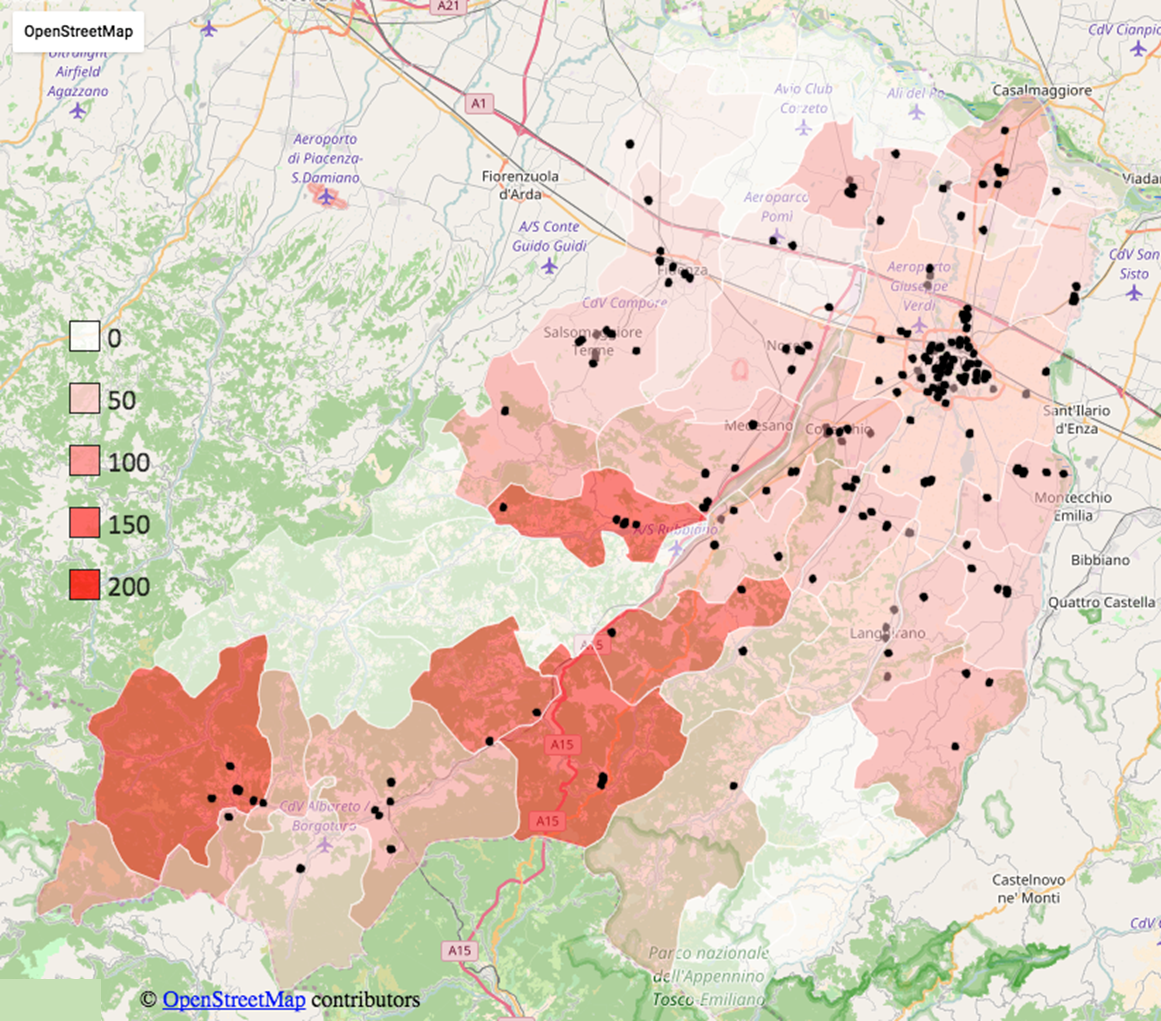

Supplement: S1 Fig — The red color scale indicates the prevalence level from zero (no cases) to 200/100.000 individuals (see S2 Table). Data from OpenStreetMap visualisation. (TIF) [file pone.0176859.s001.tif]

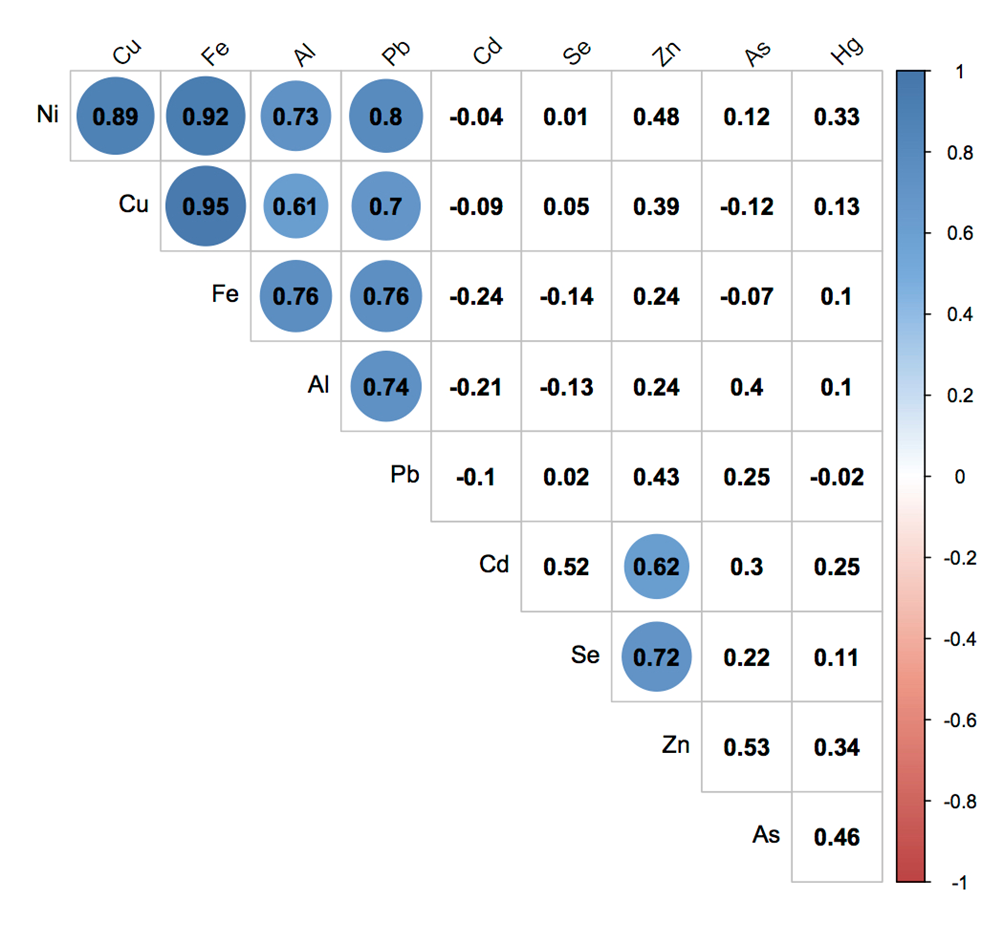

Supplement: S2 Fig — All values of Pearson correlation coefficient between elements are reported. Significant correlations are highlighted by circles (p-value <0.05). (TIF) [file pone.0176859.s002.tif]

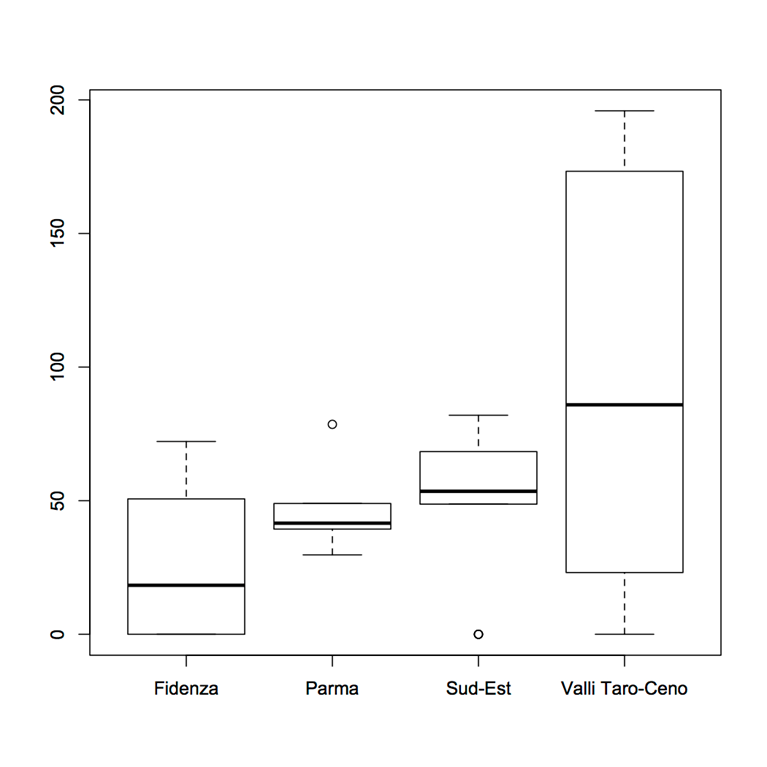

Supplement: S3 Fig — Full line shows the median value of the distirbution, lower and upper hinges correspond to the first and third quartile respectively, whiskers extend to the 95% configence interval, while data beyond that are plotted with points (outilers). (TIF) [file pone.0176859.s003.tif]

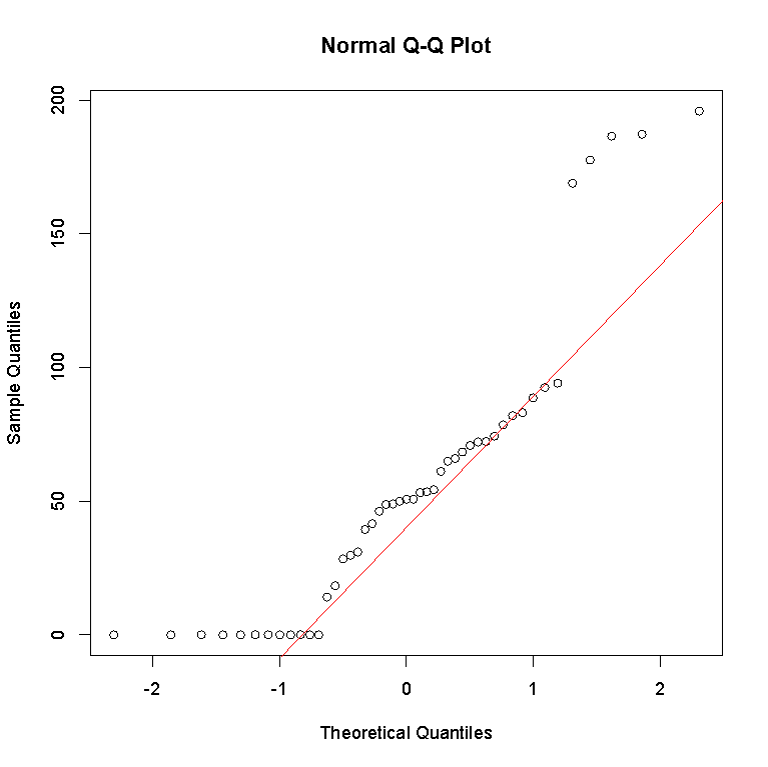

Supplement: S4 Fig — Departures from the line indicate that data are not normally distributed. (TIF) [file pone.0176859.s004.tif]

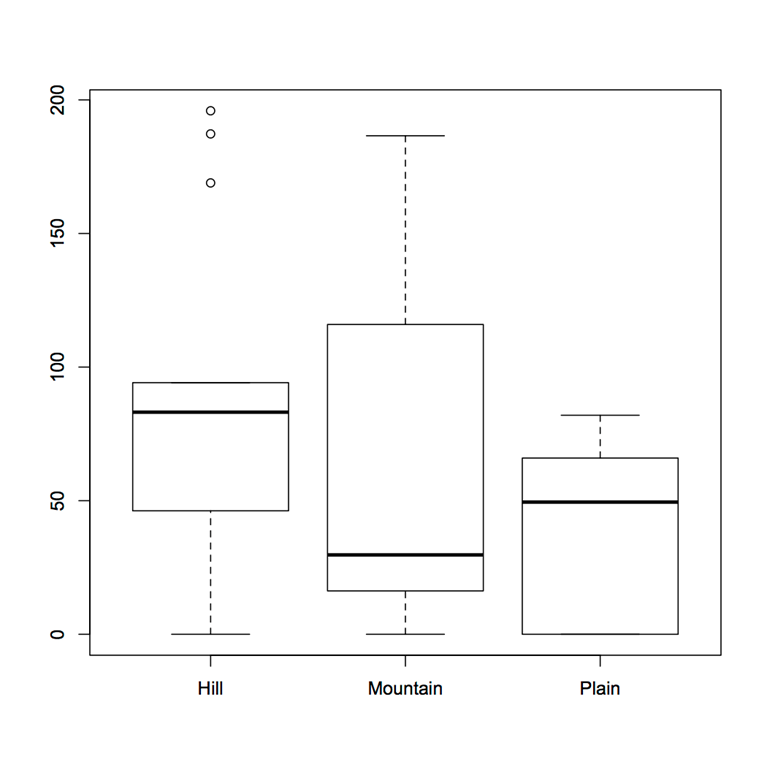

Supplement: S5 Fig — Full line shows the median value of the distirbution, lower and upper hinges correspond to the first and third quartile respectively, whiskers extend to the 95% configence interval, while data beyond that are plotted with points (outilers). (TIF) [file pone.0176859.s005.tif]

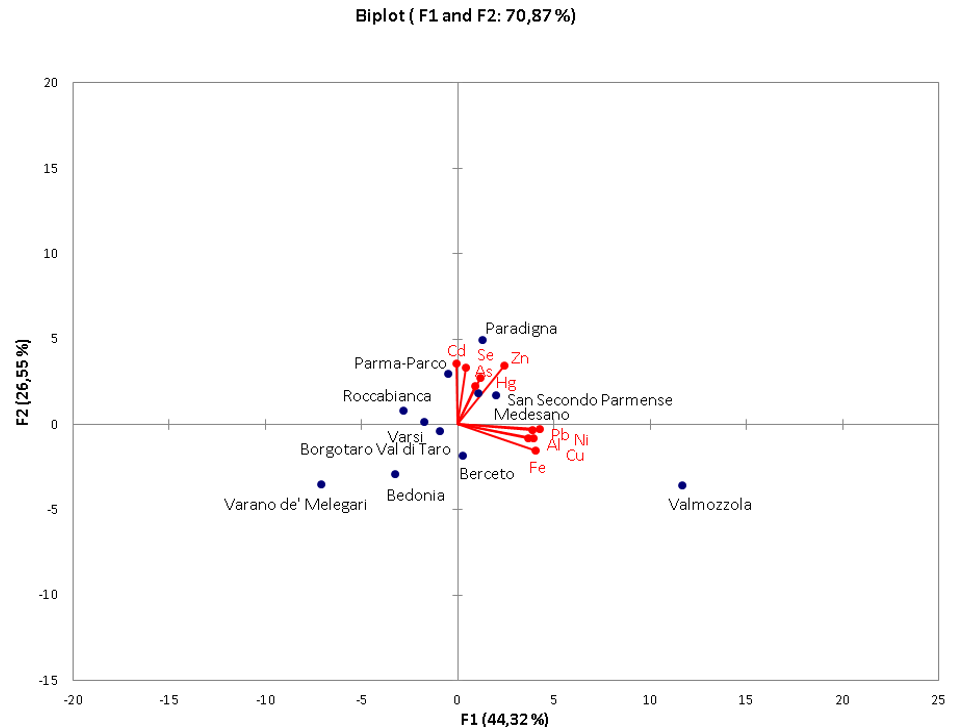

Supplement: S6 Fig — (TIF) [file pone.0176859.s006.tif]
