# Supplementary material for: Sarcoidosis in an Italian province. Prevalence and environmental risk factors
Source: PLoS One. 2017 May 5;12(5):e0176859. doi: 10.1371/journal.pone.0176859 (PMC5419555; doi:10.1371/journal.pone.0176859)
Supplement: S1 Text — (DOCX) [file pone.0176859.s007.docx]

**Statistical Analysis of the prevalence data (Health Districts and altitude-based groups)**

The set of municipalities under study is administratively (and geographically) divided into 4 health districts (“Parma”, “Fidenza”, “Sud-Est”, and “Valli Taro e Ceno”).

In order to asses whether there are any significant differences in the data coming from the four districts, we applied both parametric and non-parametric statistical tests.

A first graphic comparison among prevalence data from the four HD is provided by the boxplot in S3 Fig., that allows one to check quickly for symmetry and the existence of outliers.

At a first sight, the subpopulation corresponding to “Valli Taro e Ceno” HD presents larger median and spread with respect to the other HD, with “Fidenza” HD being also characterized by lower values of prevalence.

In order to provide statistical significance to this impression, in the main text (Fig. 2) we employed the one-way ANOVA test, finding that the difference in the means of the four groups are significant (F=4.9, p=0.005). In particular, only prevalence from “Valli Taro-Ceno” HD was found to be significantly different than the rest of the population.

The assumptions behind the ANOVA test are normality of data and homoschedasticity. To verify the first assumption, we applied the Shapiro-Wilk normality test on the data under study. The test adopts the null hypothesis that a given sample of data comes from a normally distributed population, the p-value giving the probability of the observed result assuming that the null hypothesis is true. Hence, if the p-value resulting from the test is less than a given threshold level (e.g. we adopted a threshold value 0.05), the null hypothesis is rejected and there is evidence that the data are not from a normally distributed population. On the full set of data (all municipalities, no groups) the Shapiro-Wilk test gives a very low p-value (approx. 1.9x10^-5^), suggesting that no normality hypothesis could be accepted. This is also evident from the quantile-quantile plot in S4 Fig., where the comparison with a normal distribution (red line) shows the very strong deviations due to municipalities with zero prevalence but also to municipalities with very large prevalence. The same occurs when we consider data for each district (p-value < 0.05), apart from “Parma” district in which however the result of Shapiro-Wilk test is affected by the small sample size (quantile-quantile plot shows in fact two outliers out of five data).

For the assumption of homogeneity of variances, we applied the Fligner-Killeen median test [1], a non-parametric test that is robust against non normality. The test gives a very low p-value, suggesting that data variances between different groups are significantly different.

These results suggest that the underlying assumptions of the oneway ANOVA test are not completely in the data under study. Moreover, it was not possible to reobtain normality just by means of a non-linear (Box-Cox) transformations of the data (with an optimized parameter obtained from a maximum likelihood calculation). For this reason, we employed non-parametric tests to verify the validity ofthe results. In particular, Kruskal-Wallis is a non-parametric method for testing whether samples originate from the same distribution looking at the rank statistic. The null hypothesis is that the mean ranks in the different groups are the same. Obviously, when this hypothesis is rejected, it means that the distribution of data in the various groups are different. The opposite is not true, although. When applied to the dataset with groups identified by health districts, we find a moderately low p-value (p= 0.024), suggesting that the group data should come from different distributions.

Finally, we considered the one-sided Mann-Whitney-Wilcoxon non-parametric test [2], in which the null hypothesis of equality of the mean ranks of two groups is tested against the alternate hypothesis that one group mean rank is strictly greater than the other. In particular we compared each HD with the rest of the population. No comparison was significant but that between “Valli Taro-Ceno” HD and the rest, for which we found a clear signature of a tendency towards larger values in prevalence (p-value = 0.005).

We repeated the same analysis as before in the case of the three altitude-based groups (“Plain”, “Hills”, and ”Mountain”). From the boxplot representation in S5 Fig., data belonging to the Hill group look larger than for the other groups. However, a quantitative analysis with the oneway ANOVA test did not find significant differences among the groups, although the calculation presents the same issues related to non-normality and heteroschedasticity of the data. Non conclusive results are obtained also using the Kruskal-Wallis test (p-value = 0.08). By comparing directly each group with the rest of the population, using the one-sided Mann-Withney-Wilcoxon non-parametric test, we found instead a significatively larger mean rank for the Hill group (p-value = 0.015).

**References**

[1] Conover WJ, Johnson ME, Johnson MM. A comparative study of tests for homogeneity of variances, with applications to the outer continental shelf bidding data. Technometrics 1981; 23:351-361.

[2] Mann HB, Whitney DR. On a Test of Whether one of Two Random Variables is Stochastically Larger than the Other. Annals of Mathematical Statistics 1947; 18(1):50-60.
