# Supplementary material for: Sarcoidosis in an Italian province. Prevalence and environmental risk factors
Source: PLoS One. 2017 May 5;12(5):e0176859. doi: 10.1371/journal.pone.0176859 (PMC5419555; doi:10.1371/journal.pone.0176859)
Supplement: S1 Table — (DOCX) [file pone.0176859.s008.docx]

**S1 Table** Characteristics of Municipalities Districts (MDs) belonging to four Health Districts (HDs) of Parma province

| **HDs** | **MDs** | **Population** | **Square measures**  **km^2^** | **Population Density**  **inhab/km^2^** | **Altitude**  **m ASL** |
| --- | --- | --- | --- | --- | --- |
| **Valli Taro e Ceno** |  |  |  |  |  |
|  | Albareto | 2164 | 104.11 | 21 | 512 |
|  | Bardi | 2306 | 189.9 | 12 | 625 |
|  | Bedonia | 3573 | 169.56 | 21 | 500 |
|  | Berceto | 2136 | 131.71 | 16 | 808 |
|  | Bore | 789 | 43.01 | 18 | 835 |
|  | Borgo Val di Taro | 7218 | 151.49 | 48 | 411 |
|  | Compiano | 1128 | 37.53 | 30 | 520 |
|  | Fornovo di Taro | 6160 | 57.52 | 107 | 158 |
|  | Medesano | 10768 | 88.77 | 121 | 136 |
|  | Pellegrino Parmense | 1081 | 82.08 | 13 | 410 |
|  | Solignano | 1776 | 73.14 | 24 | 232 |
|  | Terenzo | 1184 | 72.7 | 16 | 541 |
|  | Tornolo | 1062 | 67.48 | 16 | 620 |
|  | Valmozzola | 563 | 67.64 | 8.32 | 565 |
|  | Varano de’ Melegari | 2680 | 64.92 | 41 | 190 |
|  | Varsi | 1269 | 80.07 | 16 | 412 |
| **Fidenza** |  |  |  |  |  |
|  | Busseto | 7080 | 76.59 | 92 | 40 |
|  | Fidenza | 25797 | 95.12 | 271 | 75 |
|  | Fontanellato | 7038 | 53.98 | 130 | 45 |
|  | Fontevivo | 5450 | 26 | 210 | 53 |
|  | Noceto | 12895 | 79.17 | 163 | 74 |
|  | Polesine Parmense | 1501 | 24.9 | 60 | 36 |
|  | Roccabianca | 3058 | 40.46 | 76 | 32 |
|  | Salsomaggiore Terme | 19735 | 81.5 | 242 | 157 |
|  | San Secondo Parmense | 5544 | 37.71 | 147 | 38 |
|  | Sissa | 4232 | 42.9 | 98.6 | 32 |
|  | Soragna | 4891 | 45.39 | 108 | 47 |
|  | Trecasali | 3761 | 29.05 | 129.5 | 33 |
|  | Zibello | 1820 | 23.62 | 77 | 35 |
| **Sud-Est** |  |  |  |  |  |
|  | Calestano | 2053 | 57.36 | 36 | 417 |
|  | Collecchio | 14110 | 58.83 | 240 | 112 |
|  | Corniglio | 1973 | 165.7 | 12 | 690 |
|  | Felino | 8776 | 38.35 | 229 | 180 |
|  | Langhirano | 10003 | 70.84 | 141 | 265 |
|  | Lesignano Bagni | 4908 | 47.49 | 103 | 252 |
|  | Monchio delle Corti | 967 | 69.04 | 14 | 820 |
|  | Montechiarugolo | 10613 | 48.2 | 220 | 128 |
|  | Neviano Arduini | 3660 | 105.96 | 35 | 517 |
|  | Palanzano | 1122 | 69.8 | 16 | 691 |
|  | Sala Baganza | 5531 | 30.76 | 180 | 176 |
|  | Tizzano Val Parma | 2103 | 78.39 | 27 | 814 |
|  | Traversetolo | 9342 | 54.86 | 170 | 170 |
| **Parma** |  |  |  |  |  |
|  | Colorno | 8910 | 48.41 | 184 | 29 |
|  | Mezzani | 3364 | 27.65 | 122 | 26 |
|  | Parma | 177714 | 260.6 | 682 | 57 |
|  | Sorbolo | 9619 | 39.33 | 245 | 34 |
|  | Torrile | 7622 | 37.15 | 205 | 32 |
