# Supplementary material for: Sarcoidosis in an Italian province. Prevalence and environmental risk factors
Source: PLoS One. 2017 May 5;12(5):e0176859. doi: 10.1371/journal.pone.0176859 (PMC5419555; doi:10.1371/journal.pone.0176859)
Supplement: S2 Table — (DOCX) [file pone.0176859.s009.docx]

**S2** **Table** Number of cases and prevalence of sarcoidosis in Municipalities Districts (MDs) of the Province of Parma to Health Districts (HDs) (2000-2013).

| **HDs** | **MDs** | **Cases** | **Prevalence** |
| --- | --- | --- | --- |
| **Valli Taro e Ceno** |  |  |  |
|  | Albareto | 1 | 46.21 |
|  | Bardi | 0 | 0 |
|  | Bedonia | 7 | 195.91 |
|  | Berceto | 4 | 187.27 |
|  | Bore | 0 | 0 |
|  | Borgo Val di Taro | 6 | 83.12 |
|  | Compiano | 1 | 88.65 |
|  | Fornovo di Taro | 4 | 64.935 |
|  | Medesano | 8 | 74.29 |
|  | Pellegrino Parmense | 1 | 92.51 |
|  | Solignano | 0 | 0 |
|  | Terenzo | 2 | 168.92 |
|  | Tornolo | 1 | 94.16 |
|  | Valmozzola | 1 | 177.62 |
|  | Varano de’ Melegari | 5 | 186.57 |
|  | Varsi | 0 | 0 |
| **Fidenza** |  |  |  |
|  | Busseto | 1 | 14.12 |
|  | Fidenza | 8 | 31.01 |
|  | Fontanellato | 2 | 28.42 |
|  | Fontevivo | 1 | 18.35 |
|  | Noceto | 7 | 54.28 |
|  | Polesine Parmense | 0 | 0 |
|  | Roccabianca | 0 | 0 |
|  | Salsomaggiore Terme | 10 | 50.67 |
|  | San Secondo Parmense | 4 | 72.15 |
|  | Sissa | 0 | 0 |
|  | Soragna | 0 | 0 |
|  | Trecasali | 2 | 53.18 |
|  | Zibello | 0 | 0 |
| **Sud-Est** |  |  |  |
|  | Calestano | 1 | 48.71 |
|  | Collecchio | 10 | 70.87 |
|  | Corniglio | 1 | 50.68 |
|  | Felino | 6 | 68.37 |
|  | Langhirano | 5 | 49.98 |
|  | Lesignano Bagni | 3 | 61.12 |
|  | Monchio delle Corti | 0 | 0 |
|  | Montechiarugolo | 7 | 65.94 |
|  | Neviano Arduini | 3 | 81.97 |
|  | Palanzano | 0 | 0 |
|  | Sala Baganza | 4 | 72.32 |
|  | Tizzano Val Parma | 0 | 0 |
|  | Traversetolo | 5 | 53.52 |
| **Parma** |  |  |  |
|  | Colorno | 7 | 78.56 |
|  | Mezzani | 1 | 29.73 |
|  | Parma | 87 | 48.95 |
|  | Sorbolo | 4 | 41.58 |
|  | Torrile | 3 | 39.36 |
