# Supplementary material for: Sarcoidosis in an Italian province. Prevalence and environmental risk factors
Source: PLoS One. 2017 May 5;12(5):e0176859. doi: 10.1371/journal.pone.0176859 (PMC5419555; doi:10.1371/journal.pone.0176859)
Supplement: S3 Table — (DOCX) [file pone.0176859.s010.docx]

**S3 Table** Variable Contribution (%) to the Principal Components (F1 and F2).

| **Metal** | **F1** | **F2** |
| --- | --- | --- |
| As | 1,662 | 14,448 |
| Al | 15,394 | 1,240 |
| Cd | 0,006 | 24,754 |
| Cu | 17,911 | 1,226 |
| Fe | 18,897 | 4,380 |
| Hg | 0,915 | 9,606 |
| Ni | 20,820 | 0,156 |
| Pb | 17,335 | 0,246 |
| Se | 0,204 | 21,245 |
| Zn | 6,855 | 22,698 |
