# Supplementary material for: Sarcoidosis in an Italian province. Prevalence and environmental risk factors
Source: PLoS One. 2017 May 5;12(5):e0176859. doi: 10.1371/journal.pone.0176859 (PMC5419555; doi:10.1371/journal.pone.0176859)
Supplement: S1 File — (PDF) [file pone.0176859.s011.pdf]

GENDER	ETHNICITY	MD	HD
M	Causasian	BUSSETO	Fidenza
M	Causasian	FIDENZA	Fidenza
F	Causasian	FIDENZA	Fidenza
M	Causasian	FIDENZA	Fidenza
F	Causasian	FIDENZA	Fidenza
F	Causasian	FIDENZA	Fidenza
M	Causasian	FIDENZA	Fidenza
M	Causasian	FIDENZA	Fidenza
M	Causasian	FIDENZA	Fidenza
F	Causasian	FONTANELLATO	Fidenza
F	Causasian	FONTANELLATO	Fidenza
M	Causasian	FONTEVIVO	Fidenza
M	Other	NOCETO	Fidenza
M	Causasian	NOCETO	Fidenza
M	Causasian	NOCETO	Fidenza
F	Causasian	NOCETO	Fidenza
F	Causasian	NOCETO	Fidenza
M	Causasian	NOCETO	Fidenza
M	Causasian	NOCETO	Fidenza
F	Causasian	SALSOMAGGIORE TERME	Fidenza
F	Causasian	SALSOMAGGIORE TERME	Fidenza
M	Causasian	SALSOMAGGIORE TERME	Fidenza
M	Causasian	SALSOMAGGIORE TERME	Fidenza
F	Causasian	SALSOMAGGIORE TERME	Fidenza
F	Causasian	SALSOMAGGIORE TERME	Fidenza
F	Causasian	SALSOMAGGIORE TERME	Fidenza
F	Causasian	SALSOMAGGIORE TERME	Fidenza
F	Causasian	SALSOMAGGIORE TERME	Fidenza
F	Causasian	SALSOMAGGIORE TERME	Fidenza
F	Causasian	SAN SECONDO PARMENSE	Fidenza
M	Causasian	SAN SECONDO PARMENSE	Fidenza
F	Causasian	SAN SECONDO PARMENSE	Fidenza
M	Causasian	SAN SECONDO PARMENSE	Fidenza
F	Causasian	TRECASALI	Fidenza
M	Causasian	TRECASALI	Fidenza
M	Causasian	PARMA	Parma
M	Other	PARMA	Parma
F	Causasian	PARMA	Parma
F	Causasian	PARMA	Parma
M	Causasian	PARMA	Parma
F	Causasian	PARMA	Parma
F	Causasian	PARMA	Parma
M	Other	PARMA	Parma
F	Causasian	PARMA	Parma
M	Causasian	TORRILE	Parma
F	Causasian	PARMA	Parma
F	Causasian	COLORNO	Parma
M	Causasian	COLORNO	Parma

F	Causasian	COLORNO	Parma
F	Causasian	COLORNO	Parma
M	Causasian	COLORNO	Parma
F	Causasian	COLORNO	Parma
F	Causasian	COLORNO	Parma
F	Causasian	PARMA	Parma
F	Causasian	PARMA	Parma
M	Causasian	PARMA	Parma
F	Causasian	PARMA	Parma
M	Causasian	PARMA	Parma
F	Causasian	PARMA	Parma
M	Causasian	PARMA	Parma
F	Causasian	PARMA	Parma
F	Causasian	PARMA	Parma
F	Causasian	PARMA	Parma
F	Causasian	PARMA	Parma
M	Causasian	parma	Parma
F	Causasian	PARMA	Parma
F	Causasian	PARMA	Parma
F	Causasian	PARMA	Parma
F	Causasian	PARMA	Parma
F	Causasian	PARMA	Parma
F	Causasian	PARMA	Parma
F	Causasian	PARMA	Parma
F	Causasian	PARMA	Parma
F	Causasian	PARMA	Parma
F	Other	PARMA	Parma
M	Causasian	PARMA	Parma
F	Other	PARMA	Parma
F	Causasian	PARMA	Parma
F	Causasian	PARMA	Parma
F	Causasian	PARMA	Parma
M	Causasian	PARMA	Parma
F	Causasian	PARMA	Parma
F	Causasian	PARMA	Parma
F	Causasian	PARMA	Parma
F	Causasian	PARMA	Parma
F	Causasian	PARMA	Parma
F	Causasian	PARMA	Parma
F	Causasian	PARMA	Parma
F	Causasian	PARMA	Parma
M	Causasian	PARMA	Parma
F	Causasian	PARMA	Parma
F	Causasian	PARMA	Parma
F	Causasian	PARMA	Parma
F	Other	PARMA	Parma
F	Other	PARMA	Parma
F	Causasian	PARMA	Parma
F	Causasian	PARMA	Parma
M	Causasian	PARMA	Parma

F	Causasian	PARMA	Parma
M	Causasian	PARMA	Parma
M	Causasian	PARMA	Parma
F	Causasian	PARMA	Parma
M	Causasian	PARMA	Parma
M	Causasian	PARMA	Parma
F	Causasian	PARMA	Parma
F	Causasian	PARMA	Parma
F	Causasian	PARMA	Parma
M	Causasian	PARMA	Parma
F	Causasian	PARMA	Parma
F	Causasian	PARMA	Parma
F	Causasian	PARMA	Parma
M	Causasian	PARMA	Parma
M	Causasian	PARMA	Parma
F	Causasian	PARMA	Parma
M	Causasian	PARMA	Parma
F	Causasian	PARMA	Parma
M	Causasian	PARMA	Parma
F	Causasian	PARMA	Parma
M	Causasian	PARMA	Parma
F	Causasian	PARMA	Parma
M	Causasian	PARMA	Parma
F	Causasian	PARMA	Parma
F	Causasian	PARMA	Parma
F	Causasian	PARMA	Parma
F	Causasian	PARMA	Parma
M	Causasian	PARMA	Parma
F	Causasian	PARMA	Parma
F	Causasian	PARMA	Parma
M	Other	PARMA	Parma
M	Other	PARMA	Parma
F	Other	PARMA	Parma
M	Causasian	SORBOLO	Parma
M	Causasian	SORBOLO	Parma
M	Causasian	SORBOLO	Parma
F	Causasian	SORBOLO	Parma
F	Other	TORRILE	Parma
M	Causasian	TORRILE	Parma
F	Causasian	COLLECCHIO	Sud-Est
F	Causasian	SALABAGANZA	Sud-Est
M	Causasian	CALESTANO	Sud-Est
F	Causasian	COLLECCHIO	Sud-Est
F	Causasian	COLLECCHIO	Sud-ESt
F	Causasian	COLLECCHIO	Sud-Est
F	Causasian	COLLECCHIO	Sud-Est
M	Causasian	COLLECCHIO	Sud-Est
M	Causasian	COLLECCHIO	Sud-Est
M	Other	COLLECCHIO	Sud-Est
M	Causasian	COLLECCHIO	Sud-Est

M	Causasian	COLLECCHIO	Sud-Est
F	Causasian	CORNIGLIO	Sud-Est
M	Causasian	FELINO	Sud-Est
M	Causasian	FELINO	Sud-Est
F	Causasian	FELINO	Sud-Est
M	Causasian	FELINO	Sud-Est
F	Causasian	FELINO	Sud-Est
M	Causasian	LANGHIRANO	Sud-Est
M	Causasian	LANGHIRANO	Sud-Est
M	Causasian	LANGHIRANO	Sud-ESt
F	Causasian	LANGHIRANO	Sud-Est
F	Causasian	LANGHIRANO	Sud-Est
F	Causasian	lesignano bagni	Sud-Est
F	Causasian	lesignano bagni	Sud-Est
M	Causasian	lesignano bagni	Sud-Est
F	Causasian	MONTECHIARUGOLO	Sud-Est
F	Causasian	MONTECHIARUGOLO	Sud-Est
M	Causasian	MONTECHIARUGOLO	Sud-Est
M	Causasian	MONTECHIARUGOLO	Sud-Est
F	Causasian	MONTECHIARUGOLO	Sud-Est
M	Causasian	MONTECHIARUGOLO	Sud-Est
M	Causasian	MONTECHIARUGOLO	Sud-Est
F	Causasian	NEVIANO DEGLI ARDUINI	Sud-Est
M	Causasian	NEVIANO DEGLI ARDUINI	Sud-Est
F	Causasian	NEVIANO DEGLI ARDUINI	Sud-Est
M	Causasian	SALA BAGANZA	Sud-Est
M	Causasian	SALA BAGANZA	Sud-Est
F	Other	SALA BAGANZA	Sud-Est
F	Causasian	TRAVERSETOLO	Sud-Est
F	Causasian	TRAVERSETOLO	Sud-Est
M	Other	TRAVERSETOLO	Sud-Est
F	Causasian	TRAVERSETOLO	Sud-Est
M	Other	FELINO	Sud-Est
F	Causasian	TRAVERSETOLO	Sud-Est
M	Causasian	VALMOZZOLA	Val taro e Ceno
M	Causasian	MEDESANO	Valli Taro e Ceno
M	Causasian	ALBARETO	Valli Taro e Ceno
M	Causasian	BEDONIA	Valli Taro e Ceno
M	Causasian	BEDONIA	Valli Taro e Ceno
F	Causasian	BEDONIA	Valli Taro e Ceno
M	Causasian	BEDONIA	Valli Taro e Ceno
M	Causasian	BEDONIA	Valli Taro e Ceno
M	Causasian	BERCETO	Valli Taro e Ceno
F	Causasian	BERCETO	Valli Taro e Ceno
M	Causasian	BERCETO	Valli Taro e Ceno
M	Causasian	BERCETO	Valli Taro e Ceno
M	Causasian	BORGO VAL DI TARO	Valli Taro e Ceno
F	Causasian	BORGO VAL DI TARO	Valli Taro e Ceno
M	Causasian	BORGO VAL DI TARO	Valli Taro e Ceno
M	Causasian	BORGO VAL DI TARO	Valli Taro e Ceno

F	Causasian	COMPIANO	Valli Taro e Ceno
F	Causasian	FORNOVO DI TARO	Valli Taro e Ceno
F	Causasian	FORNOVO DI TARO	Valli Taro e Ceno
F	Causasian	FORNOVO DI TARO	Valli Taro e Ceno
M	Causasian	FORNOVO DI TARO	Valli Taro e Ceno
F	Causasian	MEDESANO	Valli Taro e Ceno
F	Causasian	MEDESANO	Valli Taro e Ceno
F	Causasian	MEDESANO	Valli Taro e Ceno
M	Causasian	MEDESANO	Valli Taro e Ceno
F	Causasian	MEDESANO	Valli Taro e Ceno
F	Causasian	MEDESANO	Valli Taro e Ceno
F	Causasian	PELLEGRINO PARMENSE	Valli Taro e Ceno
F	Causasian	MEDESANO	Valli Taro e Ceno
M	Causasian	TERENZO	Valli Taro e Ceno
F	Causasian	TERENZO	Valli Taro e Ceno
M	Causasian	TORNOLO	Valli Taro e Ceno
F	Causasian	VARANO DE' MELEGARI	Valli Taro e Ceno
F	Causasian	VARANO DE' MELEGARI	Valli Taro e Ceno
F	Causasian	VARANO DE' MELEGARI	Valli Taro e Ceno
M	Causasian	VARANO DE' MELEGARI	Valli Taro e Ceno
F	Causasian	VARANO DE' MELEGARI	Valli Taro e Ceno
M	Causasian	Bedonia	Valli Taro e Ceno
M	Causasian	Bedonia	Valli Taro e Ceno
M	Causasian	BORGO VAL DI TARO	Valli Taro e Ceno
F	Causasian	BORGO VAL DI TARO	Valli Taro e Ceno
