# Supplementary material for: Sarcoidosis in an Italian province. Prevalence and environmental risk factors
Source: PLoS One. 2017 May 5;12(5):e0176859. doi: 10.1371/journal.pone.0176859 (PMC5419555; doi:10.1371/journal.pone.0176859)
Supplement: S2 File — (PDF) [file pone.0176859.s012.pdf]

Site		Trace metals (mg Kg^{-1} dry wt.)				
Id	MD	As	Al	Cd	Cr	Cu
1	Paradigna	0,165 ± 0,07	417,45 ± 53,85964	0,094 ± 0,042927	n.q. ± /	8,71 ± 2,011895
2	Parma-Parco	0,179 ± 0,07	400,4 ± 51,98508	0,056 ± 0,027647	n.q. ± /	11,89 ± 2,620758
3	Varano	0,012 ± 0,01	251,35 ± 35,00233	0,01 ± 0,006398	n.q. ± /	2,76 ± 0,757901
4	Ramiola	0,216 ± 0,09	524,7 ± 65,40703	0,046 ± 0,023392	n.q. ± /	7,04 ± 1,679086
5	Varsi	0,197 ± 0,08	403,15 ± 52,28823	0,01 ± 0,006398	n.q. ± /	4,85 ± 1,223482
6	San Secondo Parmense	0,188 ± 0,08	465,85 ± 59,12009	0,019 ± 0,011037	n.q. ± /	7,48 ± 1,767826
7	Roccabianca	0,145 ± 0,06	278,85 ± 38,22983	0,067 ± 0,032197	n.q. ± /	5,66 ± 1,395011
8	Berceto	0,185 ± 0,08	536,8 ± 66,68616	0,01 ± 0,006398	n.q. ± /	4,66 ± 1,182643
9	Borgo Val di Taro	0,207 ± 0,08	609,4 ± 74,27363	0,041 ± 0,021214	n.q. ± /	3,89 ± 1,01443
10	Valmozzola	0,111 ± 0,05	718,3 ± 85,40668	0,01 ± 0,006398	n.q. ± /	31,86 ± 6,054347
11	Bedonia	0,093 ± 0,04	416,9 ± 53,79936	0,01 ± 0,006398	n.q. ± /	5,15 ± 1,287479

Fe	Hg	Mn	Ni	Pb	Se	Zn
10,12 ± 2,285393	0,125 ± 0,054687	n.q. ± /	3,41 ± 0,907058	1,518 ± 0,45609	0,209 ± 0,08463	47,157 ± 8,44768
10,67 ± 2,390483	0,1 ± 0,045244	n.q. ± /	1,716 ± 0,506154	1,034 ± 0,329151	0,187 ± 0,077	42,185 ± 7,684786
5,72 ± 1,407563	0,057 ± 0,028066	n.q. ± /	0,836 ± 0,274773	0,407 ± 0,149077	0,088 ± 0,040588	11,726 ± 2,590018
12,76 ± 2,782788	0,133 ± 0,057646	n.q. ± /	3,938 ± 1,025054	1,474 ± 0,444835	0,088 ± 0,040588	41,195 ± 7,531308
9,13 ± 2,094016	0,165 ± 0,069233	n.q. ± /	2,255 ± 0,638349	1,045 ± 0,332123	0,088 ± 0,040588	23,617 ± 4,694771
11,11 ± 2,473968	0,056 ± 0,027647	n.q. ± /	2,75 ± 0,755567	1,628 ± 0,484017	0,205 ± 0,083114	45,848 ± 8,248057
6,16 ± 1,499025	0,056 ± 0,027647	n.q. ± /	1,639 ± 0,486794	1,342 ± 0,410758	0,088 ± 0,040588	34,496 ± 6,477308
11,99 ± 2,639471	0,05 ± 0,025109	n.q. ± /	2,31 ± 0,651551	1,925 ± 0,558064	0,088 ± 0,040588	24,277 ± 4,805993
11,66 ± 2,577629	0,065 ± 0,031378	n.q. ± /	1,749 ± 0,514411	1,1 ± 0,346915	0,088 ± 0,040588	25,003 ± 4,927814
46,75 ± 8,385703	0,082 ± 0,038225	n.q. ± /	7,678 ± 1,8075	2,497 ± 0,696093	0,088 ± 0,040588	36,949 ± 6,866548
7,59 ± 1,789886	0,036 ± 0,018995	n.q. ± /	1,012 ± 0,323192	1,353 ± 0,413616	0,088 ± 0,040588	18,986 ± 3,900221
